# Discovery of Encrypted Peptides in a Human Matrix Metallopeptidase

**DOI:** 10.1021/jacsau.5c00947

**Published:** 2025-12-10

**Authors:** Rosa Gaglione, Martina Schibeci, Erika Piccolo, Rosanna Culurciello, Carla Zannella, Francesca Mensitieri, Fabrizio Dal Piaz, Valeria Cafaro, Anna De Filippis, Elio Pizzo, Eugenio Notomista, Marcelo D T Torres, Cesar de la Fuente-Nunez, Angela Arciello

**Affiliations:** † Department of Chemical Sciences, University of Naples Federico II, Via Vicinale Cupa Cintia, 26, 80126 Naples, Italy; ‡ Istituto Nazionale di Biostrutture e Biosistemi (INBB), Via dei Carpegna, 19, 00165 Rome, Italy; § Department of Biology, University of Naples Federico II, Via Vicinale Cupa Cintia, 26, 80126 Naples, Italy; ∥ Department of Experimental Medicine, University of Campania “Luigi Vanvitelli”, 80138 Naples, Italy; ⊥ Department of Medicine, Surgery and Dentistry, University of Salerno, 84084 Fisciano, Italy; # Machine Biology Group, Departments of Psychiatry and Microbiology, Institute for Biomedical Informatics, Institute for Translational Medicine and Therapeutics, Perelman School of Medicine, University of Pennsylvania, Philadelphia, Pennsylvania 19104, United States; ∇ Departments of Bioengineering and Chemical and Biomolecular Engineering, School of Engineering and Applied Science, University of Pennsylvania, Philadelphia, Pennsylvania 19104, United States; ○ Department of Chemistry, School of Arts and Sciences, University of Pennsylvania, Philadelphia, Pennsylvania 19104, United States; ◆ Penn Institute for Computational Science, University of Pennsylvania, Philadelphia, Pennsylvania 19104, United States

**Keywords:** encrypted peptides, antimicrobial peptides, human matrix metallopeptidase-19, antibiotic resistance, drug discovery, anti-infective activity

## Abstract

The human proteome represents a vast, largely untapped source of encrypted bioactive peptides with therapeutic potential. Here, we report the discovery and functional characterization of three antimicrobial encrypted peptides (EPs) derived from human matrix metallopeptidase-19 (residues 1-19, 1-33, and 247-279). These peptides exhibit potent, broad-spectrum activity against Gram-positive and Gram-negative bacteria, including clinical isolates and multidrug-resistant strains. Mechanistic studies reveal membrane depolarization and permeabilization as the primary mechanism of action. The peptides also inhibit biofilm formation, eradicate preformed biofilms, and exhibit selective antiviral activity against enveloped viruses. Importantly, they display negligible hemolysis and cytotoxicity toward mammalian cells while modulating inflammation through LPS neutralization. Synergy assays reveal synergistic or additive interactions with last-line antibiotics, and no resistance emerged after serial bacterial passaging. A fully d-amino acid analog of the lead peptide retained activity and exhibited cytocompatibility and *in vivo* efficacy in a murine skin infection model. These findings underscore the therapeutic promise of human protein-derived encrypted peptides and highlight proteome mining as a viable strategy for identifying host-compatible anti-infectives.

## Graphical abstract

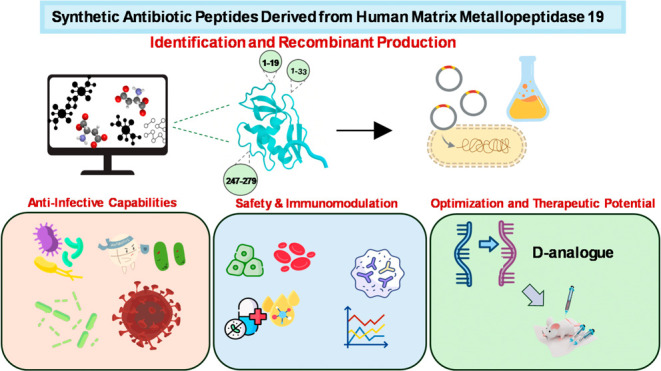

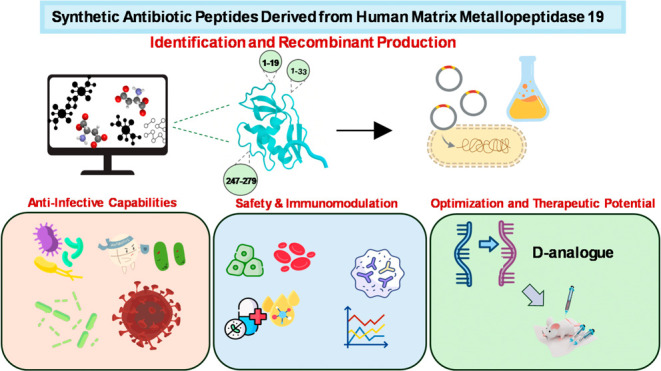

## Introduction

The swift rise of antibiotic-resistant bacteria poses a major and escalating threat to modern medicine.^1–3^ Multidrug-resistant (MDR) pathogens have become some of the most challenging agents of hospital-acquired infections, significantly reducing the number of effective therapeutic options available.^4,5^ The widespread misuse and overuse of antibiotics in both clinical and agricultural settings have accelerated the emergence of bacteria resistant to all known drug classes, so-called pan-drug-resistant (PDR) strains, which were responsible for over 4 million deaths in 2021 alone.^6^ While the dissemination of microbial resistance genes is a primary driver of treatment failure, it is increasingly evident that complex host-related factors, such as immune dysregulation, tissue damage, and systemic inflammation, also contribute to poor clinical outcomes in infections like sepsis.^7–10^

In response to the urgent need for alternatives to traditional antibiotics, antimicrobial peptides (AMPs) have gained considerable attention. AMPs are typically short (fewer than 50 amino acid residues), amphipathic peptides that can target a wide range of pathogens, including bacteria, fungi, and viruses.^11–18^ These peptides possess a vast sequence diversity and often act through mechanisms that are not easily circumvented by resistance.^2,19^ Several AMP candidates have entered clinical trials; however, their development is often hindered by challenges related to cytotoxicity, proteolytic instability, and manufacturing costs.^20–22^

Recent advances in computational biology are beginning to transform the landscape of AMP discovery. Machine learning, deep learning, generative algorithms, and pattern recognition techniques are being employed to design novel AMP sequences with enhanced efficacy and reduced off-target effects.^23–33^ Despite these advances, relatively few studies have tapped into the enormous potential of proteome- and metagenome-derived AMP discovery, especially from nonimmune proteins that may harbor encrypted bioactive fragments.^12,27,28,33–38^ This approach offers the possibility of uncovering functionally active peptides embedded within larger proteins, which may have evolved for unrelated biological roles but become antimicrobial upon proteolytic processing.

It is now evident that eukaryotic proteins not primarily involved in immunity can yield encrypted bioactive fragments after proteolysis.^38–40^ Our group developed a scoring algorithm that integrates charge, hydrophobicity and peptide length to predict antibacterial potency.^41–43^ This approach facilitated the exploration of a previously uncharted region of peptide sequence space, resulting in the identification of 43,000 candidate peptides, 2,603 of which were predicted to exhibit antimicrobial activity.^38,41,42^

Although matrix metallopeptidase-19 (MMP-19) is conventionally recognized for its role in extracellular matrix remodeling, it has been implicated in key inflammatory and innate immune processes. It is highly expressed in the epidermis and upregulated under inflammatory conditions, where it contributes to proper T-cell–mediated cutaneous immune responses and T-cell distribution.^43^ MMP-19 also plays a critical role in regulating host responses to colonic pathogens and in coordinating innate immunity in mouse models of colitis, supporting mucosal healing and tissue homeostasis.^44–49^ Based on these observations, we hypothesize that it may also contribute directly to innate immunity through proteolytic processing events that generate antimicrobial peptide fragments. Using our bioinformatic platform, we pinpointed two AMP-like regions within human MMP-19. They encompass residues 1-33 (with local maxima at 1-19 and 1-33) and 247-279. We produced three recombinant peptides, r­(P)­YLL19, r­(P)­YLL33, and r­(P)­PRT33, via fusion to onconase in *Escherichia coli*, purified them at 4–6 mg·L^–1^ yields and confirmed the peptide identity by mass spectrometry (MS). In this study, we detail the antimicrobial, anti-biofilm, antiviral, immunomodulatory, and *in vivo* properties of these peptides and describe a fully D-enantiomeric analogue with enhanced proteolytic stability. Our results demonstrate the untapped potential of human proteins as reservoirs of cryptic antimicrobial activity and underscore the value of proteome mining for next-generation peptide therapeutics.

## Results and Discussion

### Identification and Production of MMP-19-Derived Encrypted Peptides

Numerous precursor proteins harboring encrypted peptides (EPs) with biological functions unrelated to those of the parent protein have been discovered throughout the human body, offering an alternative source for antibiotic discovery.^14,38–40,42,50^ Notable examples are hemoglobin, thrombin, lactoferrin, lysozyme, histone-like proteins and vertebrate secretory ribonucleases.^51–53^ Using an algorithmic approach that scores physicochemical features characteristic of AMPs,^41^ we identified two encrypted regions within human MMP-19 (Figure 1A and Supporting Figures S1 and S2). The first region spans residues 1-33 and contains two local maxima: residues 1-33 (absolute score, AS = 13.3) and residues 1-19 (AS = 10.8). The two-dimensional plots showing AS values as a function of position for selected window lengths (19 and 33 residues) are reported in Supporting Figure S1. The second region extends from A242 to V279 (AS ≈ 12-13), with a local maximum at residues 247-279 (AS = 12.6). The two-dimensional plot showing AS values as a function of position for the selected window length (33 residues) is reported in Supporting Figure S2. To the best of our knowledge, this is the first report identifying MMP-19 as a source of AMPs. We recombinantly produced three peptides: r­(P)­YLL19 (residues 1-19), r­(P)­YLL33 (residues 1-33), and r­(P)­PRT33 (residues 247-279) (Figure 1A). To this purpose, *E. coli* BL21­(DE3) cells were transformed with pET22b­(+) recombinant plasmids encoding each peptide fused to the carrier protein onconase, following previously established cost-effective production procedures.^14,50^ Acidic cleavage at the Asp–Pro bond released the peptides, leaving a Pro residue at the N-terminus (Figure 1B). Notably, this artificial addition of an N-terminal proline, introduced for production purposes, is not expected to significantly affect the antimicrobial activity of the peptides. This has been experimentally confirmed for several peptides identified using the same bioinformatic approach in apolipoprotein E and human fibrinogen, where both synthetic and recombinant forms exhibited essentially identical antimicrobial activity.^54,55^ In the case of r­(P)­PRT33 peptide, the presence of an internal Asp–Lys acid-labile bond necessitated an additional RP-HPLC step to separate the full-length peptide from two cleavage fragments: r­(P)­PRT12 (residues 1-12) and KVW21 (residues 13-33) (Supporting Figures S3 and S4). Mass spectrometric analysis confirmed the identity of all purified peptides (Supporting Table S1). Final yields were 4.5 mg L^–1^ for r­(P)­YLL19, 6.5 mg L^–1^ for r­(P)­YLL33, and 6 mg L^–1^ for r­(P)­PRT33.

**Figure 1:**
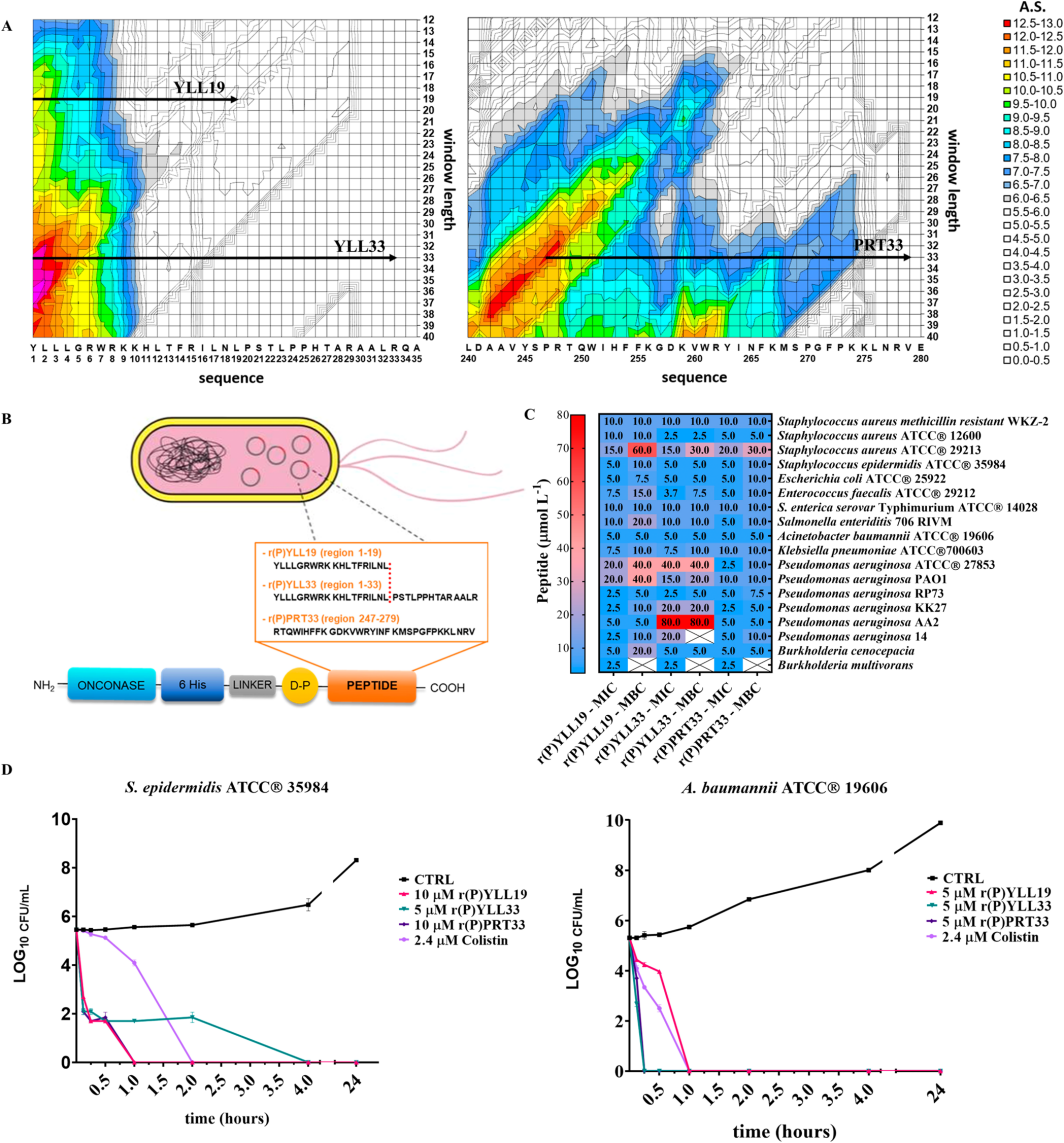
Identification, production and antimicrobial activity of MMP-19-derived encrypted peptides. (A) Novel antimicrobial peptides were identified within the MMP-19 precursor protein through a previously reported *in silico* prediction strategy.^41^ All computational tools are provided in the Supporting Information of the original publication (Files S1.xls and S2.doc).^41^ Two regions of MMP-19 were selected as a source of putative antimicrobial peptides. Two-dimensional plots showing AS values as a function of residue position for selected window lengths are provided in Supporting Figures S1 and S2. (B) Recombinant expression of peptides in bacterial cells. The first identified region (residues 1-33) was recombinantly produced in two forms: a shorter peptide (residues 1-19), termed r­(P)­YLL19, and a longer peptide (residues 1-33), termed r­(P)­YLL33. In all cases, recombinant peptides were expressed as chimeric proteins fused to the carrier protein onconase. (C) Antimicrobial activity of MMP-19-derived peptides against a panel of 18 bacterial strains. Experiments were performed using an initial bacterial inoculum of ∼2 × 10^6^ CFU mL^–1^. Reported data represent the mean ± SD from three independent experiments. (D) Time-kill curves of *S. epidermidis* ATCC 35984 and *A. baumannii* ATCC 19606 treated over 0–24 h with MMP-19-derived peptides at their respective MICs (10 μM r­(P)­YLL19, 5 μM r­(P)­YLL33, 10 μM r­(P)­PRT33 for *S. epidermidis* ATCC 35984; 5 μM r­(P)­YLL19, 5 μM r­(P)­YLL33, 5 μM r­(P)­PRT33 for *A. baumannii* ATCC 19606). Colistin (2.4 μM) was used as a positive control, while untreated cells served as negative controls.

### Antimicrobial Activity

The antimicrobial activity of MMP-19-derived EPs was assessed against 18 bacterial strains, including Gram-positive, Gram-negative, antibiotic-resistant, and clinical isolates, as shown in Figure 1C. Broth microdilution assays^56,57^ were performed to determine the minimum inhibitory (MIC) and minimum bactericidal (MBC) concentration (Figure 1C), defined as the lowest concentrations that completely inhibit bacterial growth (MIC) or kill bacterial cells (MBC). Interestingly, the peptides inhibited the growth and killed both Gram-positive and Gram-negative strains at concentrations ranging mostly between 2.5 and 40 μM, including *S. aureus* ATCC 29213, *S. aureus* MRSA WKZ-2 and *E. coli* ATCC 25922, which are common pathogens under surveillance.^58,59^ Significant antibacterial effects were observed for MMP-19-derived EPs on *S. enterica* serovar Typhimurium ATCC 14028, the clinically isolated *S. enteriditis* 706 RIVM and *E. faecalis* ATCC 29212, key foodborne pathogens. Notably, all three peptides exerted significant bacteriostatic and bactericidal effects on *Pseudomonas* and *Burkholderia* spp., which were clinically isolated from cystic fibrosis patients and exhibit “natural” antimicrobial resistance.^60,61^ While the peptides displayed antimicrobial activity against a broad range of both sensitive and resistant bacterial strains, including clinical isolates, the low MIC and MBC values observed for MMP-19-derived EPs against *A. baumannii* ATCC 19606 (Gram-negative) and *S. epidermidis* ATCC 35984 (Gram-positive) led us to select these strains as model organisms for further studies. Time-kill assays revealed that r­(P)­YLL19 eradicated both strains within 1 h (Figure 1C). r­(P)­YLL33 and r­(P)­PRT33 achieved complete killing of *A. baumannii* ATCC 19606 within 15 min (Figure 1D), while r­(P)­PRT33 and r­(P)­YLL33 required 1 and 4 h, respectively, to kill *S. epidermidis* ATCC 35984 (Figure 1D).

### Morphological Effects

Scanning electron microscopy (SEM) analyses of bacteria treated with MMP-19-derived EPs at MBC concentrations revealed progressive membrane disruption, and detachment of cell wall, which increased over time (Figure 2A). After 3 h, r­(P)­YLL19 induced membrane permeabilization, cell shrinkage, and cytoplasmic leakage in both *A. baumannii* ATCC 19606 and *S. epidermidis* ATCC 35984 bacterial strains. By 16 h, bacterial surfaces appear corrugated with dimples, and most cells were lysed and gutted (Figure 2A). r­(P)­YLL33 and r­(P)­PRT33 caused pronounced outer membrane wrinkling and fragmentation within 3 h (Figure 2A). In the case of *A. baumannii* ATCC 19606, treatment with r­(P)­PRT33 generated surface blisters, likely resulting from peptide-induced displacement of Mg^2+^ ions in the lipopolysaccharide layer, destabilizing the outer membrane and facilitating inner membrane disruption. This destabilization may enhance AMP penetration, leading to local inner membrane damage, accumulation of cytoplasmic content in the periplasmic space, and blister formation, without complete outer membrane rupture, as previously described for other AMPs.^62,63^ In light of these findings, all three MMP-19-derived EPs appear to exert their antimicrobial effects through interactions with bacterial membranes, although the morphological outcomes of this interaction vary depending on the bacterial species.

**Figure 2:**
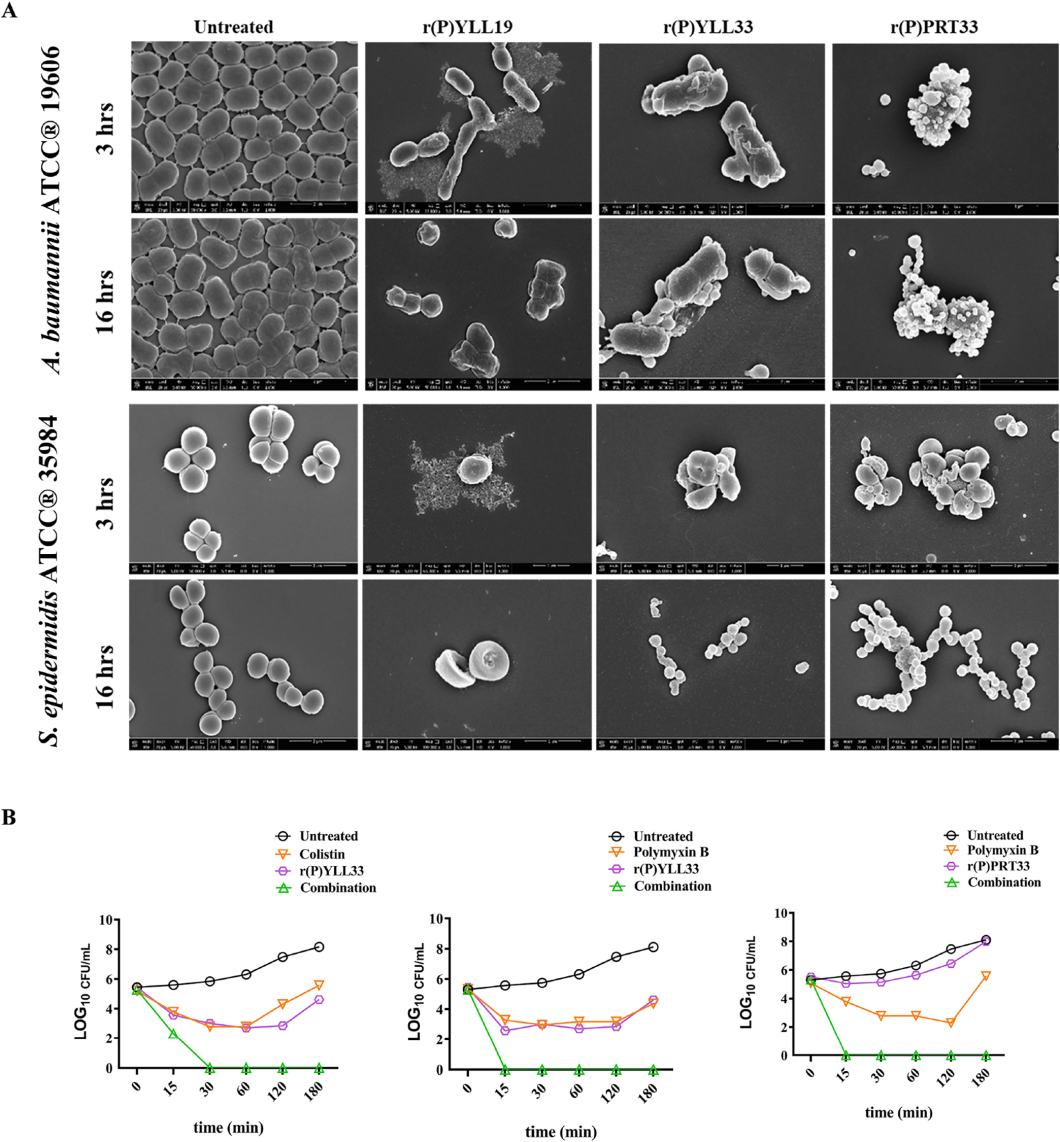
Antimicrobial activity of MMP-19-derived EPs. (A) Morphological analyses of *S. epidermidis* ATCC 35984 and *A. baumannii* ATCC 19606 by SEM. Representative images are shown. Sixty cells were analyzed *per* condition in two independent experiments. Scale bars: 1 and 2 μm. (B) Time-kill curves showing the effects of combinatorial treatments using MMP-19-derived peptides with colistin or polymyxin B on *S. epidermidis* ATCC 35984. Results are compared with single-agent treatments at equivalent sub-MIC concentrations. The initial bacterial inoculum was approximately 2 × 10^6^ CFU mL^–1^. Peptides, polymyxin B, and colistin were tested at the sub-MIC levels used in the combination assays. The first panel represents a combination with an FICI of 0.71 (2.5 μM r­(P)­YLL33 + 4 μM colistin), while the second and third panels correspond to combinations with FICI values of 1.0 (2.5 μM r­(P)­YLL33 + 2 μM polymyxin B; 2.5 μM r­(P)­PRT33 + 2 μM polymyxin B).

### Synergy with Conventional Antibiotics

Chequerboard assays were performed to evaluate the synergy between MMP-19-derived EPs and conventional antibiotics against *A. baumannii* ATCC 19606 and *S. epidermidis* ATCC 35984. In this assay, a two-dimensional array of serial concentrations of the test compounds is used to calculate the fractional inhibitory concentration index (FICI), which assesses whether drug combinations produce effects greater than the sum of their individual effects.^64^ Additive interactions (0.5 < FICI ≤ 1) were observed for r­(P)­YLL33 with colistin or polymyxin B, and for r­(P)­PRT33 with colistin, against the Gram-negative pathogen *A. baumannii* ATCC 19606 (Table 1). Bacterial cells were then treated with the most promising combinations, and colony-forming units were counted post-treatment. Results are shown in Supporting Figure S5, showing a markedly enhanced antibacterial effect of the peptide-antibiotic combinations compared to either agent alone. Notably, treatment of *S. epidermidis* ATCC 35984 with r­(P)­YLL33 combined with colistin or polymyxin B, as wells as r­(P)­PRT33 combined with colistin, resulted in >99.9 % bacterial cell death within 15 min, far faster than either agent alone (Figure 2B). Single-agent experiments (peptide or colistin alone) were performed at the same sub-MIC concentrations used in the combination assays, rather than at their full MICs as in Figure 1D, and thus partial bacterial regrowth was observed, as expected (Figure 2B). These findings highlight the potential of these peptides to enhance antibiotic efficacy, lower required doses and curb the development of resistance. Collectively, our data identify MMP-19 as a novel source of encrypted AMPs and underscore the therapeutic promise of its derived peptides, both as standalone agents and as enhancers of existing antibiotics.

**Table 1:** FICI determined by Performing Checkerboard Assays

		**average FICI**	
		**r(P)YLL33**	**r(P)PRT33**
*A. baumannii* **ATCC 19606**	**Colistin**	0.71	1.0
	**Polymyxin B**	1.0	4.0
	**Ciprofloxacin**	2.0	2.0
*S. epidermidis* **ATCC 35984**	**Colistin**	4.0	4.0
	**Polymyxin B**	2.0	2.0
	**Ciprofloxacin**	3.0	4.0

### Membrane Depolarization and Resistance Propensity

To evaluate whether the mechanism of action of MMP-19-derived EPs involves depolarization of bacterial membranes, we used the voltage-sensitive dye DiSC_3_(5) (3,3′-Dipropylthiadicarbocyanine Iodide). This cationic, membrane-permeable fluorophore accumulates in polarized cells, where self-quenching reduces fluorescence. Upon membrane depolarization, the dye is released, resulting in a measurable fluorescence increase. Bacterial cells were preloaded with DiSC_3_(5) and then exposed to each peptide for 1 h; the percentage of depolarization is shown in Figure 3A. All three peptides caused a significant fluorescence increase in both*A. baumannii*ATCC 19606 (Gram-negative) and *S. epidermidis* ATCC 35984 (Gram-positive), indicating substantial membrane depolarization.

**Figure 3:**
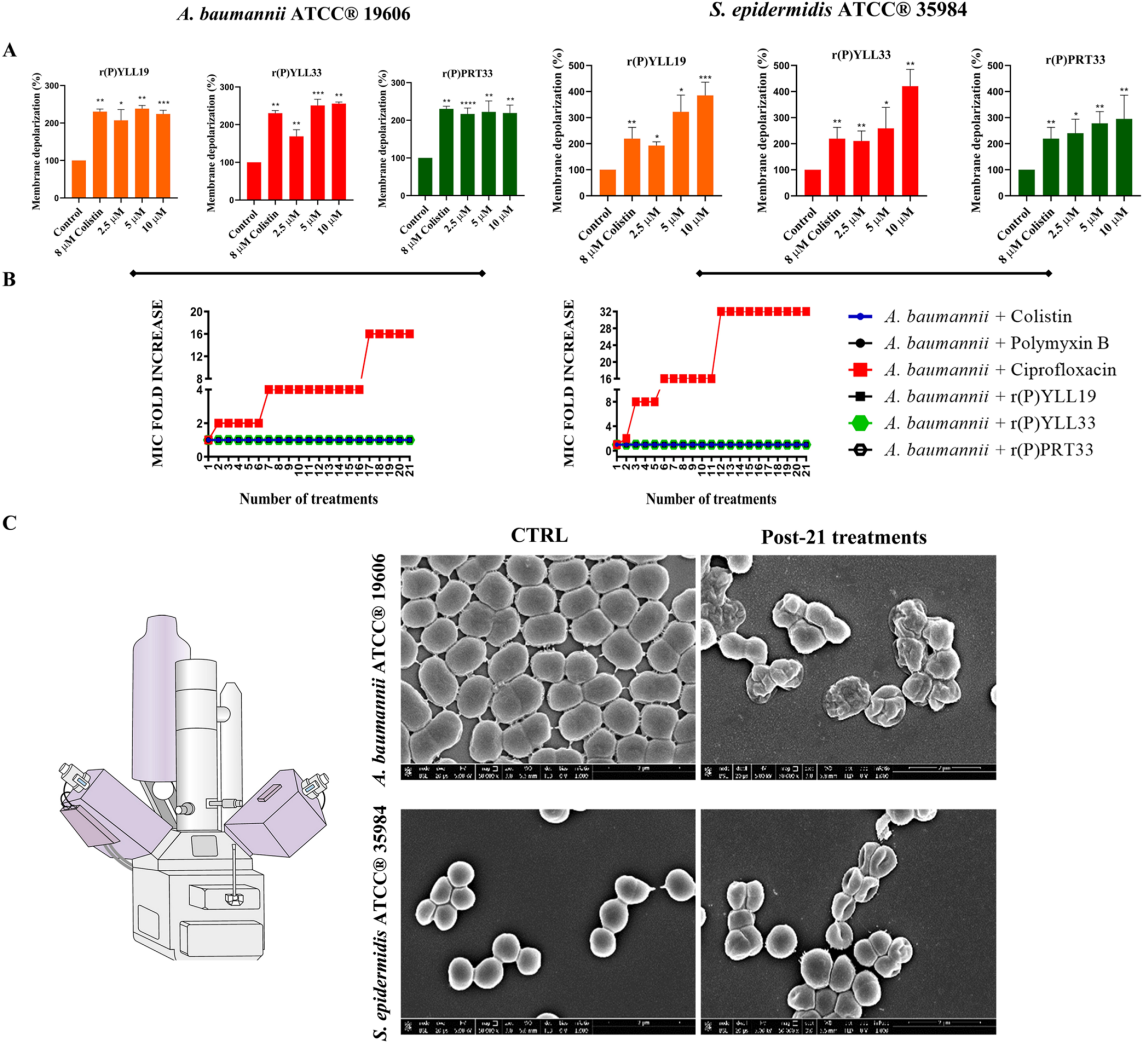
MMP-19-derived EPs affect membrane polarization without promoting resistance development. (A) Membrane depolarization in response to increasing concentrations of MMP-19-derived peptides, assessed *via* fluorescence changes of the membrane potential-sensitive dye DiSC_3_(5). Bacterial cells were treated for 1 h with peptides at 2.5, 5, and 10 μM. Colistin (8 μM) was used as a positive control, and untreated cells served as negative controls. Bacteria were incubated at an optical density (OD_600 nm_) of 0.03–0.06, corresponding to approximately 5 × 10^7^ CFU mL^–1^. Data represent the mean ± SD from three independent experiments. Statistical significance: * *p* < 0.05, ** *p* < 0.01, *** *p* < 0.001. (B) Assessment of resistance phenotype development in *A. baumannii* ATCC 19606 and *S. epidermidis* ATCC 35984 following serial exposure to colistin (4 or 8 μM), polymyxin B (2 or 4 μM), ciprofloxacin (1.5 or 6 μM), r­(P)­YLL19 (5 μM), r­(P)­YLL33 (5 μM), or r­(P)­PRT33 (5 μM). The initial bacterial inoculum was approximately 2 × 10^6^ CFU mL^–1^ in all cases. (C) SEM analysis of *A. baumannii* ATCC 19606 and *S. epidermidis* ATCC 35984 cells after 21 serial treatments with ciprofloxacin, compared to untreated control cells. Bacteria were incubated at an optical density (OD_600 nm_) of 0.1, corresponding to approximately 1 × 10^8^ CFU mL^–1^. Scale bars: 2 μm.

To assess whether the peptides interact electrostatically with bacterial surfaces, we measured the Zeta potential (ζ) after incubating *A. baumannii* ATCC 19606 and *S. epidermidis* ATCC 35984 with r­(P)­YLL19, r­(P)­YLL33, or r­(P)­PRT33 at their respective MICs for 15, 30, and 60 min. As shown in Supporting Figure S6, *A. baumannii* ATCC 19606 exhibited a modest ζ shift after 60 min, particularly after treatment with r­(P)­YLL33 and r­(P)­PRT33, whereas *S. epidermidis* ATCC 35984 showed no significant change. Thus, under these conditions, the peptides do not substantially alter the cell surface charge. This is consistent with previous reports indicating that many AMPs disrupt membranes without altering surface charge, instead inducing curvature or forming pores.^65,66^ This mechanism may also apply to the peptides examined in this study, which appear to trigger bacterial cell lysis, as observed through SEM analysis of treated cells in Figure 2A, without significantly altering surface charge.

Because conventional antibiotics often drive the development of resistance,^67,68^ whereas AMPs rarely do, we examined whether prolonged exposure to the peptides selects for resistance. *A. baumannii* ATCC 19606 and *S. epidermidis* ATCC 35984 were serially passaged in medium containing r­(P)­YLL19, r­(P)­YLL33, or r­(P)­PRT33 at their subinhibitory concentrations (MIC/2). For comparison, cells were exposed to colistin and polymyxin B (both membrane-active) or ciprofloxacin (which targets DNA gyrase/topoisomerase IV).^69,70^ Baseline MIC and MBC values for the antibiotics are provided in Supporting Table S2. MICs were redetermined, and results are summarized in Figure 3B and Supporting Tables S3 and S4. Throughout the 21 days of exposure, MICs for all three peptides, as well as for colistin and polymyxin B, remained unchanged in both species. In contrast, ciprofloxacin MICs increased 16-fold (from 6 μM to 96 μM) in *A. baumannii* ATCC 19606 and 32-fold (from 1.5 μM to 48 μM) in *S. epidermidis* ATCC 35984 (Supporting Tables S3 and S4).

SEM analyses of the ciprofloxacin-resistant isolates revealed corrugated, rumpled cell surfaces, in contrast to the smooth morphology of untreated controls (Figure 3C). Similar structural alterations have been associated with fluoroquinolone resistance and may reflect adaptive modifications that limit drug uptake or enhance efflux.^71,72^

In summary, prolonged exposure to the MMP-19-derived EPs did not select for resistant mutants under the conditions tested, whereas ciprofloxacin readily did so. These findings underscore the therapeutic potential of the peptides for treating multidrug-resistant infections without promoting further resistance.

### Anti-Biofilm Activity

Bacterial biofilms shield pathogens from antibiotics and immune responses, thereby fostering persistent infections and antimicrobial resistance. To evaluate the anti-biofilm properties of the MMP-19-derived EPs, we selected six representative strains: *S. aureus* ATCC 29213, *S. aureus* MRSA WKZ-2, *S*. *enteritidis* 706 RIVM, *S. enterica* serovar Typhimurium ATCC 14028, *S. epidermidis* ATCC 35984, and *A. baumannii* ATCC 19606. Crystal-violet assays were used to assess the effects of the peptides on the three principal stages of biofilm development: (i) surface attachment, (ii) biofilm formation/maturation, and (iii) detachment, following the protocol of Gaglione et al.^14^

All three peptides inhibited biofilm attachment at concentrations below the MBCs determined for the corresponding planktonic cells (Supporting Figure S7). The strongest inhibition was observed with *A. baumannii* ATCC 19606 (∼70% inhibition at 1.3 μM), *S. enterica* serovar Typhimurium ATCC 14028 (∼65% at 2.5 μM), and *S. aureus* MRSA WKZ-2 (∼54% at 5 μM). A similar pattern was observed for biofilm formation, except that r­(P)­PRT33 did not affect biofilm formation by the *Staphylococcus* spp. (Supporting Figure S7).

Peptide performance against preformed biofilms varied. r­(P)­YLL19 was the most effective, detaching ∼ 50% of the biofilm produced by *S. aureus* MRSA WKZ-2, *S. enteritidis* 706 RIVM, and *A. baumannii* ATCC 19606 at 20 μM (Supporting Figure S7). r­(P)­YLL33 and r­(P)­PRT33 were most active against *S. epidermidis* ATCC 35984 and *S. enteritidis* 706 RIVM at the same concentration, eradicating ∼60 and ∼20% of the biofilm, respectively (Supporting Figure S7). These data highlight the potential biomedical utility of the peptides against established biofilms.

To visualize the effects of the peptides, confocal laser scanning microscopy (CLSM) analyses were performed on *S. epidermidis* ATCC 35984 (Gram-positive) and *A. baumannii* ATCC 19606 (Gram-negative). Cultures were treated with 2.5 μM r­(P)­YLL19, r­(P)­YLL33, or r­(P)­PRT33 for 16 and 24 h at 37 °C under static conditions. CLSM revealed profound alterations in the three-dimensional architecture and volume of nascent biofilms (Figure 4, left panels). In *S. epidermidis* ATCC 35984, peptide treatment interfered with initial attachment: the signal from dead cells (red) decreased relative to controls, and biofilm thickness remained unchanged, consistent with a direct antimicrobial effect on early adherent cells. During biofilm formation, all peptides markedly reduced total biofilm volume (Figure 4, left panels).

**Figure 4:**
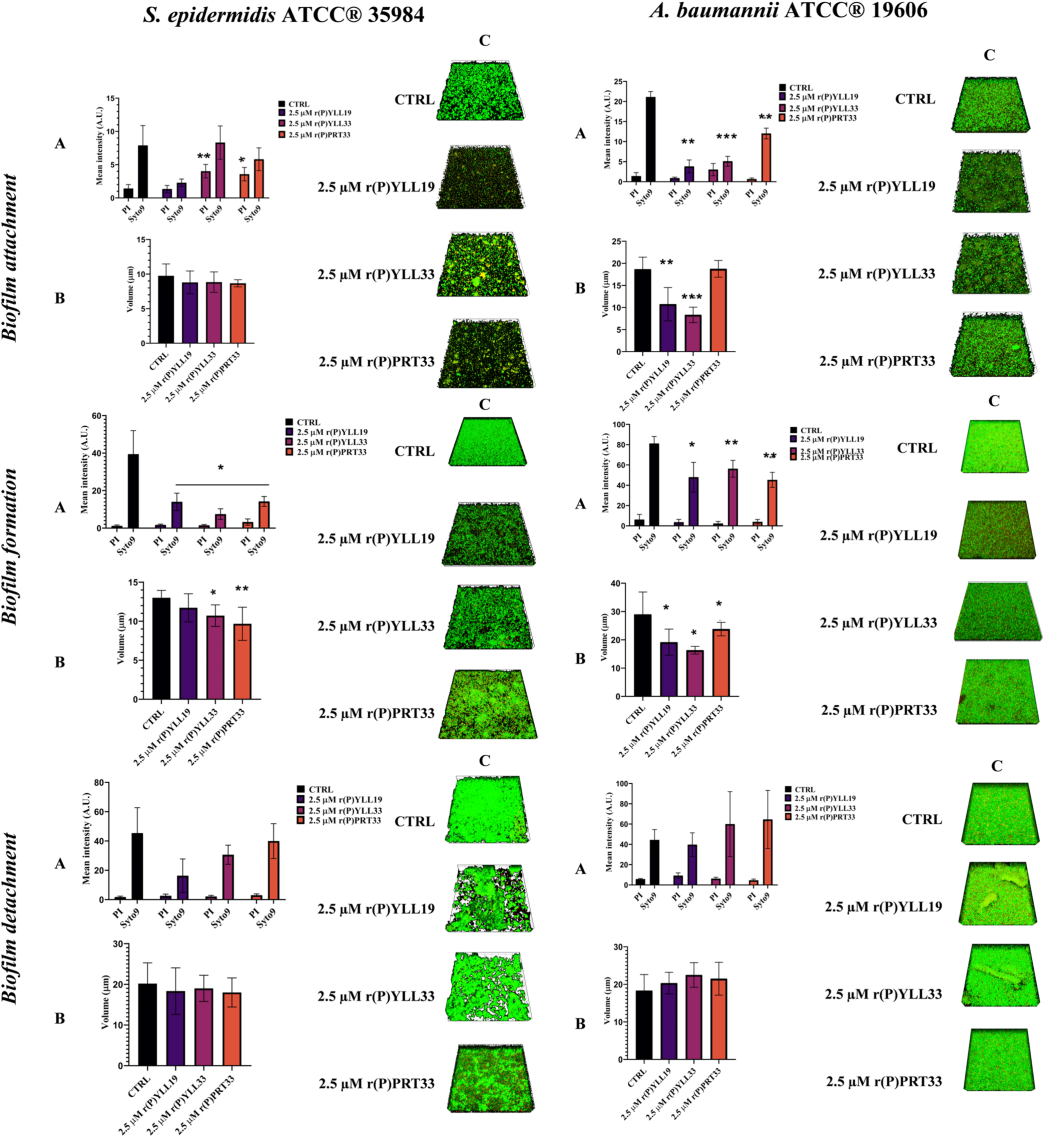
Anti-biofilm activity of MMP-19-derived EPs. Effects of MMP-19 derived peptides on *S. epidermidis* ATCC 35984 (left panels) and *A. baumannii* ATCC 19606 (right panels) biofilm attachment, formation and detachment, as analyzed by CLSM. Bacteria were treated with sub-MIC concentrations of the peptides and stained with Syto9 (total cells) and Propidium Iodide (PI; dead cells). (A) Fluorescence main intensity reported in arbitrary units, (B) biofilm thickness, and (C) representative 3D reconstructions. Data refer to three independent experiments, each comprising at least three image acquisitions. Statistical significance was determined using Student’s *t*-test, with comparisons made against the corresponding control groups. Significance levels are indicated as follows: * *p* < 0.05, ** *p* < 0.01, *** or *p* < 0.001.

More pronounced effects were observed with *A. baumannii* ATCC 19606 (Figure 4, right panels). Peptide exposure significantly disrupted both attachment and formation stages, resulting in thinner, more loosely organized biofilms. In contrast, preformed *A. baumannii* ATCC 19606 biofilms showed little reduction in overall biovolume, although notable structural disorganization was evident, especially for *S. epidermidis* ATCC 35984, suggesting partial matrix destabilization without complete biomass removal (Figure 4, right panels).

Collectively, these findings demonstrate that the MMP-19-derived EPs can hinder biofilm initiation and maturation and, to a lesser extent, destabilize established biofilms in clinically relevant Gram-positive and Gram-negative pathogens.

### Biofilm Composition Analysis

To deepen our understanding of the peptides’ anti-biofilm activity, we examined whether they alter the biochemical composition of established biofilms. *S. epidermidis* ATCC 35984 and *A. baumannii* ATCC 19606 were cultivated under biofilm-promoting conditions and treated with sublethal concentrations of each peptide. Total polysaccharides and proteins in the biofilm matrix were quantified by Dubois and Bradford assays, respectively. As shown in Supporting Figure S8, treatment with any of the three peptides significantly reduced both components in both species. Sugar content declined by ∼80% in *S. epidermidis* ATCC 35984 and ∼50% in *A. baumannii* ATCC 19606 relative to untreated controls. Peptides r­(P)­YLL19 and r­(P)­PRT33 also lowered protein content by ∼50–60% under all conditions tested. These data suggest that, upon treatment with the peptides, an alteration in the composition of the extracellular polymeric substance (EPS) occurs, as reported for several EPS-targeting AMPs.^73–75^

### Antiviral Properties

Because MMP-19-derived EPs primarily act on membranes, we next assessed their activity on enveloped viruses. After confirming a lack of cytotoxicity toward Vero-76 cells (Supporting Figure S9), we evaluated r­(P)­YLL19 and r­(P)­YLL33 in four assay formats (co-treatment, virus pretreatment, cell pretreatment, and post-treatment) using plaque reduction as the read-out. These assays enabled us to assess the peptides’ ability to interfere with various stages of the viral life cycle, including attachment, entry, and replication. Three viral models were employed: (1) Human coronavirus 229E (HCoV-229E), enveloped, (+)­ssRNA; (2) Herpes simplex virus 1 (HSV-1), enveloped, dsDNA; (3) Coxsackievirus B3 (CVB3), nonenveloped, (+)­ssRNA.

### HCoV-229E

In co-treatment assays, where Vero-76 cell monolayers were simultaneously exposed to the virus and peptide, r­(P)­YLL19 and r­(P)­YLL33 showed IC_50_ values of 12.5 and 27.8 μM, respectively (Figure 5A). Potency increased markedly in virus-pretreatment assays, where the virus was incubated with each peptide prior to infection, yielding IC_50_ values of 0.56 μM for r­(P)­YLL19 and 16.4 μM for r­(P)­YLL33 (Figure 5B), indicating direct virucidal effect. Neither peptide showed measurable activity in cell-pretreatment (Vero-76 cells were treated with peptides before infection) or post-treatment (peptides added after viral entry) formats (Figure 5C,D).

**Figure 5:**
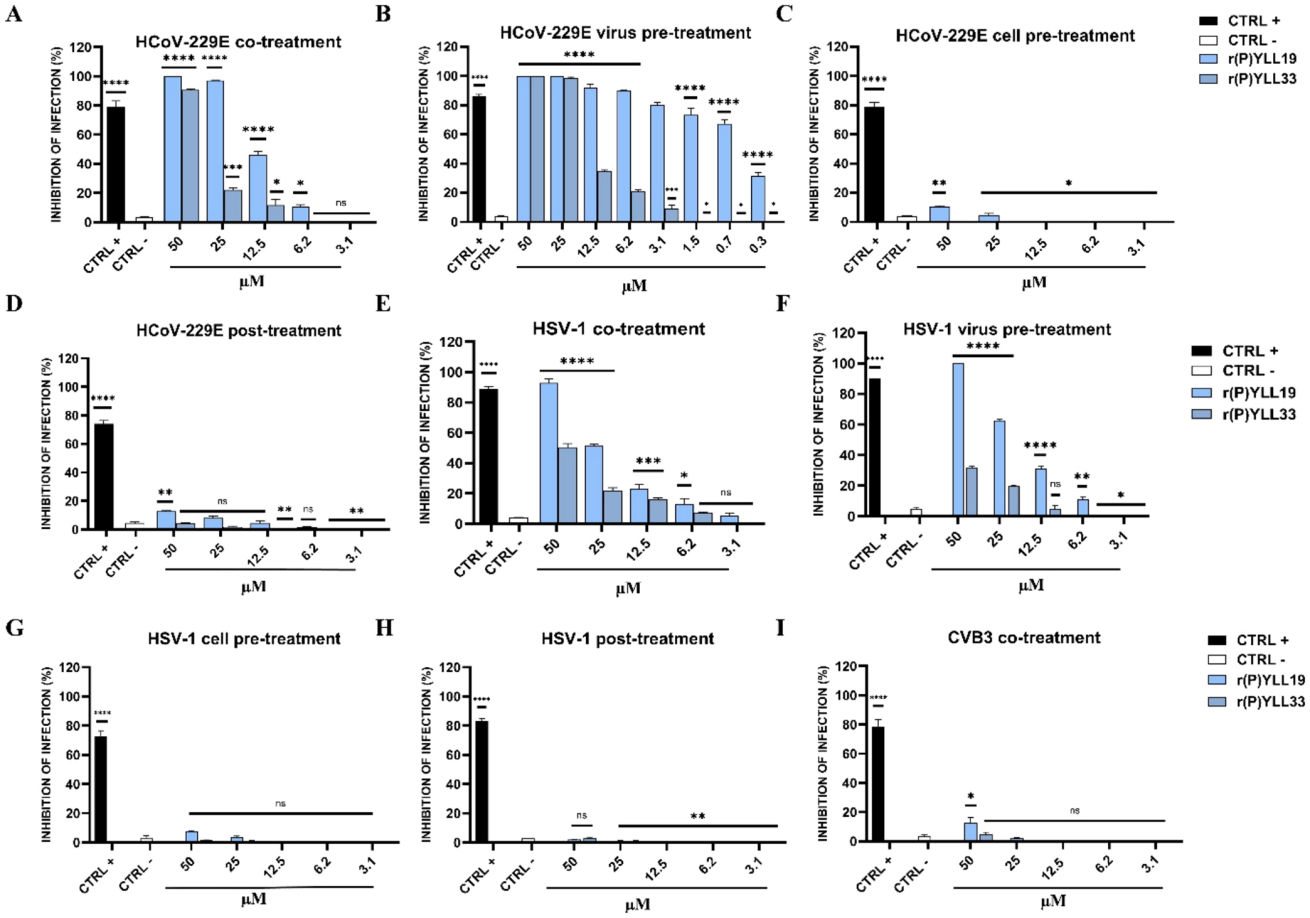
Antiviral activity of r­(P)­YLL19 and r­(P)­YLL3 peptides. (A–D) Antiviral activity against HCoV-229E under different treatment conditions: (A) co-treatment; (B) virus pretreatment; (C) cell pretreatment; (D) post-treatment. Positive control (CTRL+) refers to rhamnolipids M15RL (50 μg mL^–1^ in A and B), ivermectin (10 μM in C), and remdesivir (10 μM in D); negative control (CTRL−) refers to infected, untreated cells. (E–H) Antiviral activity against HSV-1: (E) co-treatment; (F) virus pretreatment; (G) cell pretreatment; (H) post-treatment. CTRL + refers to melittin (5 μM in E and F), dextran sulfate (1 μM in G), and aciclovir (5 μM in H); CTRL– refers to infected, untreated cells. (I) Antiviral activity against CVB3 in co-treatment assay. CTRL+ refers to pleconaril (2 μg mL^–1^); CTRL– refers to infected, untreated cells. Experiments were independently repeated three times, and data are presented as mean ± standard deviation (SD). Statistical analyses were performed using GraphPad Prism software (version 8.0.1). Significance was assessed by one-way ANOVA followed by Dunnett’s post hoc test, with differences considered statistically significant at * *p* ≤ 0.05.

### HSV-1

A similar pattern was observed, though with lower potency. r­(P)­YLL19 exhibited IC_50_ values of 25 μM (co-treatment) and 20 μM (virus pretreatment) (Figure 5E,F), while r­(P)­YLL33 displayed only marginal effects. Again, no significant activity was observed in cell-pretreatment or post-treatment assays (Figure 5G,H).

### CVB3

Results support a membrane-targeted mechanism: neither peptide inhibited the nonenveloped CVB3 in co-treatment assays (Figure 5I).

Collectively, these results suggest that r­(P)­YLL19 and r­(P)­YLL33 exert antiviral effects by directly disrupting viral envelopes during early attachment or entry steps, a biophysical mechanism that may limit resistance development and enable broad-spectrum activity against enveloped viruses. This mechanism aligns with prior studies showing that amphipathic or cationic peptides can selectively disrupt viral membranes through electrostatic and hydrophobic interactions with lipid components of the viral envelope.^76–80^ Moreover, by inhibiting early stages of viral infection, including attachment and membrane fusion, peptides may offer a strategic advantage in limiting viral propagation at the point of entry.^81^

### Biocompatibility and Immunomodulatory Properties

To evaluate the safety profile of the MMP-19-derived peptides, we first assessed their hemolytic activity against sheep red blood cells (SRBCs). Hemolysis is a known potential side effect of AMPs due to their membrane-disruptive activity, which can inadvertently damage host erythrocytes.^82^ Hemolysis was quantified by measuring hemoglobin release following peptide exposure. As shown in Figure 6A, all three peptides produced only minimal hemolysis (<5%) even at 100 μM, well above their respective MBCs, indicating a low risk of erythrocyte damage. We next examined peptide cytotoxicity toward RAW 264.7 murine macrophages, a standard model for immunological studies. Cell viability, determined by MTT reduction, remained >90% across all peptide concentrations and time points (Supporting Figure S10), confirming good cellular tolerance.

**Figure 6:**
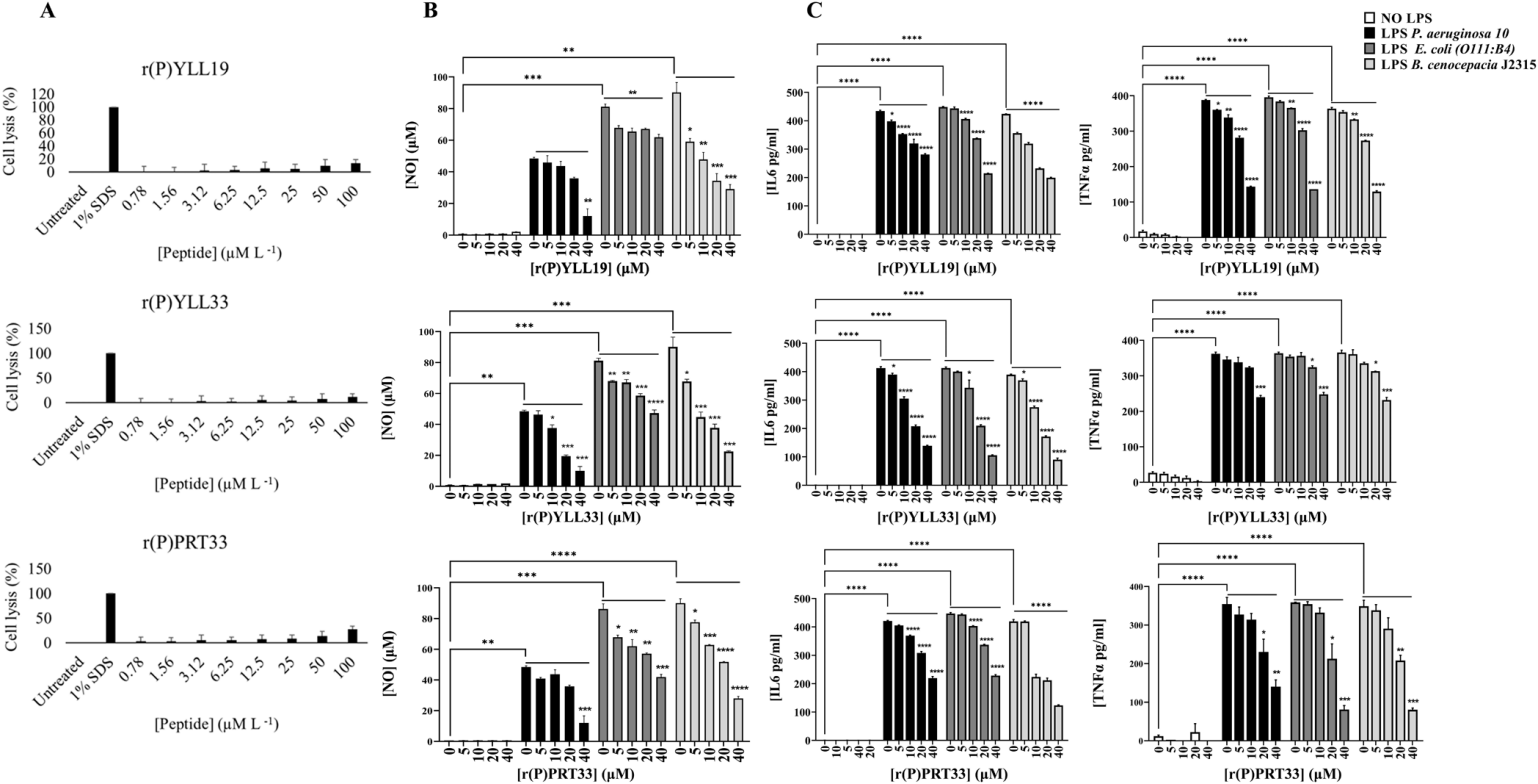
Biocompatibility and immunomodulatory properties of MMP-19-derived EPs. (A) Hemolytic effects of increasing concentrations of MMP-19-derived peptides on sheep RBCs. Controls include untreated cells and cells treated with 1% SDS (1:1 *v/v*). Data represent the mean ± standard deviation (SD) of three independent experiments. (B) Effects of r­(P)­YLL19, r­(P)­YLL33, and r­(P)­PRT33 on NO release, measured by the Griess assay. Raw 264.7 murine macrophage cells were stimulated with LPSs from *P. aeruginosa* 10, *E. coli* (O111:B4), or *B. cenocepacia* J2315 in the presence of peptides. (C) TNF-α and IL-6 release quantified by ELISA assays following LPS stimulation and peptide treatment as described in (B). All experiments were performed in triplicate. Statistical analysis was conducted using GraphPad Prism software (version 8.0.1). Significance was determined by one-way ANOVA followed by Bonferroni’s post hoc test (*p < 0.05, **p < 0.01, ***p < 0.001 or ****p < 0.0001).

### Immunomodulatory Activity

In addition to their direct antimicrobial effects, many AMPs modulate host immunity responses.^83^ To investigate this potential, RAW 264.7 cells were coincubated with lipopolysaccharide (LPS) from *Pseudomonas aeruginosa* 10, *Escherichia coli* (O111:B4), or *Burkholderia cenocepacia* J2315 along with r­(P)­YLL19, r­(P)­YLL33, or r­(P)­PRT33 (5–40 μM). After 24 h, nitric oxide (NO) release was quantified using the Griess assay (Figure 6B), overall intracellular oxidative stress was assessed using DCFH-DA and TBARS assays (Supporting Figure S11), and the release of pro-inflammatory cytokines, tumor necrosis factor-α (TNF-α) and interleukin-6 (IL-6), was measured by ELISA assays (Figure 6C). None of the peptides induced NO or cytokine release in unstimulated cells, confirming that they are not intrinsically pro-inflammatory (Figure 6C and Supporting Figure S11). Under LPS challenge, however, all three peptides significantly and dose-dependently reduced NO, TNF-α, IL-6, and oxidative-stress markers. r­(P)­YLL19 and r­(P)­PRT33 exhibited the strongest effects, lowering NO and TNF-α by ≥ 60% at 40 μM (Figure 6C and Supporting Figure S11).

These results mirror the behavior of other cationic amphipathic peptides that bind electrostatically to LPS, neutralize endotoxin activity, and attenuate inflammation without broadly suppressing immune function.^84–87^ Collectively, the MMP-19-derived peptides demonstrate excellent hemocompatibility, negligible cytotoxicity, and a desirable capacity to dampen LPS-driven inflammatory responses, attributes that support their further development as anti-infective and immunomodulatory therapeutics.

### *In Vitro* Antimicrobial Activity and Biocompatibility of the D-Enantiomeric Analogue D­(P)­YLL19

One major hurdle in peptide-drug development is the rapid degradation of peptides by proteases *in vivo*.^88^ Linear peptides are particularly susceptible, and this significantly shortens their antimicrobial lifetime.^88,89^ For example, human antimicrobial peptides derived from apolipoprotein B were completely degraded after only 30 min exposure to serum proteases (10% fetal bovine serum).^18^ Similarly, a host defense peptide identified in human 11-hydroxysteroid dehydrogenase-1 β-like protein using our bioinformatic approach was fully degraded after 24 h incubation in 50% (*v*/*v*) human serum.^90^ A well-established strategy to overcome this limitation is the substitution of native l-amino acids with their mirror-image D-residues.^91,92^ Among the MMP-19-derived candidates, r­(P)­YLL19 was selected as the lead due to its short length and outstanding anti-infective and cytocompatibility profiles (Figures 1–5). Accordingly, we synthesized the fully D-configured analogue, D­(P)­YLL19, in which each L-residue is replaced by its D-counterpart (Figure 7A).

**Figure 7:**
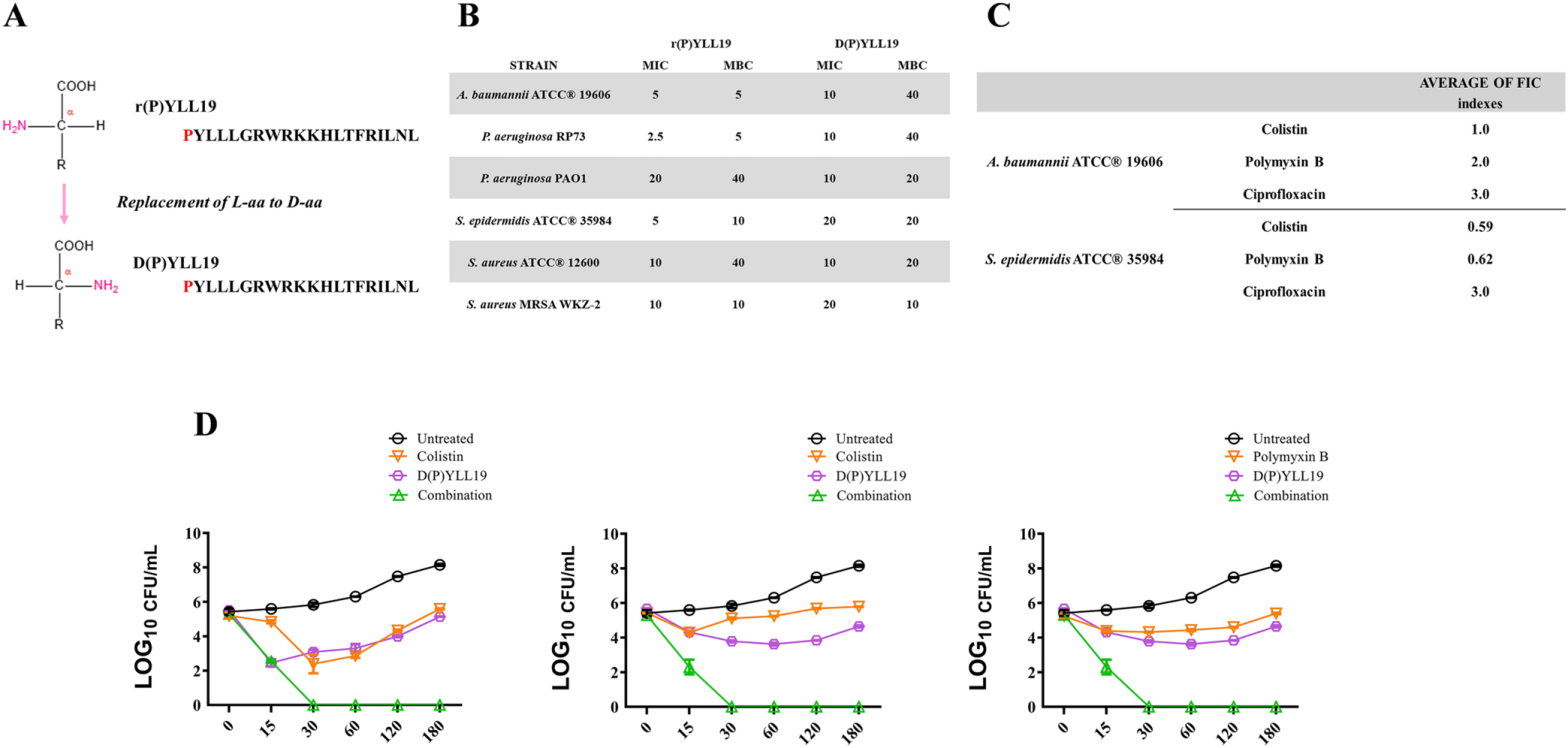
*In vitro* antimicrobial activity and biocompatibility of synthetic peptide D­(P)­YLL19. (A) Schematic representation of the design strategy showing the substitution of natural l-amino acids with D-amino acids to generate the synthetic peptide D­(P)­YLL19. (B) Antimicrobial activity of D­(P)­YLL19 against six bacterial strains. Bacterial inocula were approximately 2 × 10^6^ CFU mL^–1^. Experiments were performed in triplicate, and data are reported as mean values. MIC and MBC values of D­(P)­YLL19 were determined and compared with those of the parent peptide r­(P)­YLL19. (C) Antimicrobial activity of D­(P)­YLL19 in combination with conventional antibiotics against *S. epidermidis* ATCC 35984 and *A. baumannii* ATCC 19606. FIC indexes were determined from a minimum of three independent experiments, each performed in triplicate. (D) Time-kill kinetics of D­(P)­YLL19 in combination with colistin or polymyxin B against *A. baumannii* ATCC 19606 (first graph) and *S. epidermidis* ATCC 35984 (second and third graph). The combination of colistin and D­(P)­YLL19 corresponds to an FIC index of 1.0 against *A. baumannii* ATCC 19606 and 0.59 against *S. epidermidis* ATCC 35984; the combination of polymyxin B and D­(P)­YLL19 corresponds to an FIC index of 0.62 against *S. epidermidis* ATCC 35984.

### Antimicrobial Efficacy

D­(P)­YLL19 retained potent activity against all six test pathogens (Figure 7B), with MIC and MBC values only slightly higher than those of its L-form (compare Figures 1C and 7B). Synergy assays demonstrated strikingly enhanced bactericidal activity when the peptide was combined with colistin against *A. baumannii* ATCC 19606, or with colistin or polymyxin B against *S. epidermidis* ATCC 35984 (Figure 7C). Plate counts confirmed the superior killing effect of the combinations compared to the treatment with single agents (Supporting Figure S12), and time–kill assays showed complete bacterial eradication within 15 min (Figure 7D).

### Biocompatibility

As d-amino acid substitution can occasionally increase cytotoxicity,^92,93^ we evaluated the safety of D­(P)­YLL19 in several mammalian cell lines, including human primary dermal fibroblasts (HDF), human immortalized keratinocytes (HaCaT), human epidermoid carcinoma cells (A431), and murine macrophages (RAW 264.7). Cell viability remained above 70% even at the highest tested concentrations after 72 h of incubation. Only mild cytotoxic effects (25–30% reduction in viability) were observed for HDF and HaCaT cells at the highest peptide concentrations (Supporting Figure S13). Additionally, the peptide exhibited no hemolytic activity against sheep erythrocytes (Supporting Figure S14).

D­(P)­YLL19 thus retains the antimicrobial potency of r­(P)­YLL19 while offering markedly improved proteolytic stability and a good safety profile. D-enantiomerization is a widely used approach to enhance peptide half-life,^93^ often maintaining or even boosting antimicrobial activity while minimizing host toxicity.^94,95^ Because membrane-disruptive mechanisms are generally independent of chirality, D-peptides typically retain broad-spectrum activity.^96^ These results illustrate how d-amino-acid substitution can yield next-generation antimicrobial peptides with superior pharmacokinetic properties and therapeutic indices.

### *In Vivo* Efficacy

Before performing *in vivo* analyses, we assessed the antibacterial activity of the MMP-19-derived EPs in rich medium (Luria–Bertani, LB), where r­(P)­YLL19, r­(P)­YLL33, and r­(P)­PRT33 exhibited MICs of 64 μM, whereas D­(P)­YLL19 showed a lower MIC of 32 μM against *A. baumannii* ATCC 19606. LB broth was selected over MHB for this analysis because it contains a broader range of proteins, lipids, and metabolites, providing a richer environment that more closely reflects the physiological and clinical conditions in which these peptides are expected to act.^12,26,27,32,40,41^ Based on these findings, we assessed the peptides’ anti-infective potential *in vivo* using a murine skin abscess infection model (Figure 8). Female CD-1 mice were selected for the analysis to maintain consistency with previously published wound infection and topical treatment models, in which female mice exhibit slightly faster or more consistent wound closure kinetics compared to males.^97–99^ This selection contributed to a reduction in experimental variability. However, it should be noted that sex can influence healing responses and antimicrobial outcomes; therefore, future studies will include both sexes to assess potential sex-related differences in treatment efficacy. In the present experiments, mice were infected with *A. baumannii* ATCC 19606 and treated 2 h postinfection with a single topical dose of each peptide at its respective MIC: 64 μM for r­(P)­YLL19, r­(P)­YLL33, and r­(P)­PRT33, and 32 μM for D­(P)­YLL19. Polymyxin B (8 μM) served as a positive control.

**Figure 8:**
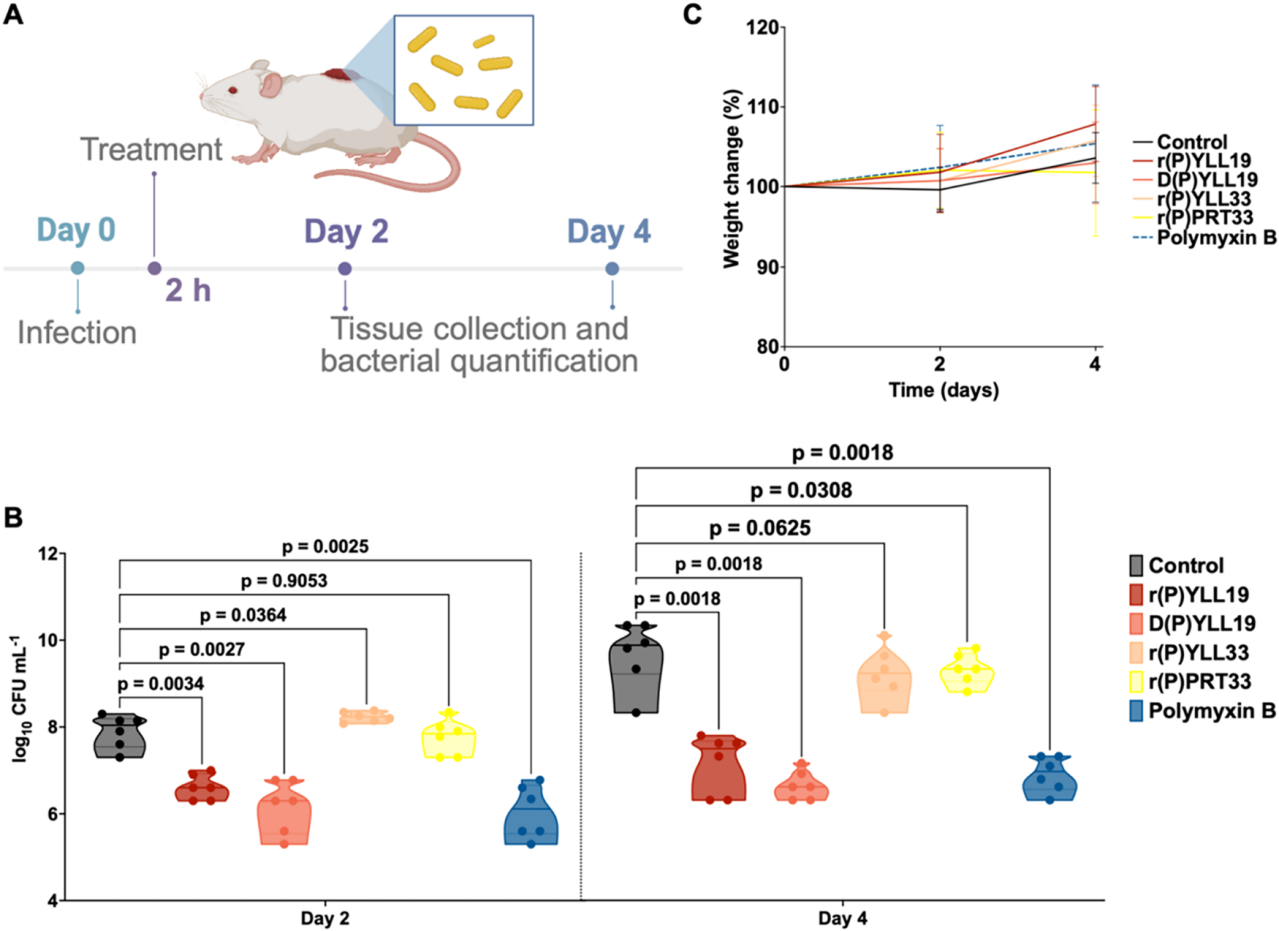
*In vivo* anti-infective activity of MMP-19-derived EPs. (A) Schematic representation of the murine skin abscess mouse model used to evaluate the anti-infective activity of MMP-19-derived peptides against *A. baumannii* ATCC 19606. (B) Peptides r­(P)­YLL19, D­(P)­YLL19, r­(P)­YLL33, and r­(P)­PRT33 were administered topically at their respective MICs in a single dose, two h postinfection. r­(P)­YLL19 and D­(P)­YLL19 significantly reduced bacterial loads by ∼3 to 4 orders of magnitude after 4 days, comparable to the effect of polymyxin B. (C) Mouse body weight was monitored throughout the 4-day experiment to evaluate potential toxicity associated with the bacterial infection and peptide treatments. Statistical analysis in panel (B) was performed using one-way ANOVA followed by Dunnett’s post hoc test. All treatment groups were compared to the untreated control; *p*-values are indicated. Violin plots represent the median, upper, and lower quartiles. Panel (A) was created using BioRender.

Treatment with r­(P)­YLL33 or r­(P)­PRT33 did not significantly reduce *A. baumannii* ATCC 19606 colony-forming unit (CFU) counts at either 2 or 4 days post-treatment. In contrast, the shorter peptides, r­(P)­YLL19 and D­(P)­YLL19, produced a substantial reduction in bacterial burden, approximately 3 to 4 orders of magnitude after 4 days, comparable to polymyxin B (Figure 8; *p* < 0.002 for D­(P)­YLL19 and r­(P)­YLL19). Notably, D­(P)­YLL19 appeared more effective than its L-form counterpart at day 2 (*p* = 0.0027 vs *p* = 0.0364), though their activities converged by day 4. Bacterial burden was quantified by homogenizing the infected skin tissue and performing CFU count assays, providing a direct measure of viable bacteria at the infection site. No significant weight loss or signs of inflammation were observed in any treatment group, indicating the absence of systemic toxicity.

These findings confirm the potent *in vivo* efficacy of both r­(P)­YLL19 and D­(P)­YLL19 and underscore their therapeutic potential. The comparable efficacy of both enantiomers supports a stereochemistry-independent, membrane-targeted mechanism, a hallmark of many effective AMPs.^100^ This observation aligns with previous reports in which D-enantiomerization preserved or enhanced antimicrobial activity. Indeed, D-peptides retained potent activity against both planktonic and biofilm-associated bacteria and demonstrated enhanced *in vivo* efficacy, effectively protecting against *P. aeruginosa* infections in animal models.^101^

The success of both versions of the peptide in our study highlights their stability, bioavailability, and potential to resist proteolytic degradation. Collectively, these findings reinforce the concept that D-enantiomeric peptide therapeutics can provide potent, resistance-evading interventions. The *in vivo* performance of both r­(P)­YLL19 and D­(P)­YLL19 supports their further development as effective antimicrobial therapeutics.

## Methods

### Materials

Unless specified otherwise, all reagents used in the present study were purchased from Sigma-Merck (Milan, Italy).

### Bacterial Strains and Growth Conditions

All bacterial strains used in the analyses [*i*.*e*., *A. baumannii* ATCC 19606, *B. cenocepacia* LMG 18863, *B. multivorans* LMG 17582, *E. faecalis* ATCC 29212, *E. coli* ATCC 25922, *K. pneumoniae* ATCC 700603, *S. aureus* ATCC 12600, *P. aeruginosa* 14, *P. aeruginosa* AA2, *P. aeruginosa* ATCC 27853, *P. aeruginosa* KK27,*P. aeruginosa* PA01, *P. aeruginosa* RP73, *S. enteriditis*RIVM 706, *S. enterica* serovar Typhimurium ATCC 14028, *S. aureus* ATCC 29213, methicillin-resistant *S. aureus* MRSA WKZ-2, and *S. epidermidis* ATCC 35984] were grown in the same media and experimental conditions as previously reported.^14,16,42,102^ Bacterial strains *B. cenocepacia* LMG 18863, *B. multivorans* LMG 17582, *P. aeruginosa* 14, *P. aeruginosa* AA2, *P. aeruginosa* KK 27, and *P. aeruginosa* RP 73 were kindly provided by Dr. Alessandra Bragonzi (Infection and CF Unit, San Raffaele Scientific Institute, Milan, Italy).

### Peptides

Expression and isolation of recombinant peptides were carried out as previously described.^18,103^ Briefly, following recombinant expression, each fusion protein was purified by affinity chromatography using Ni Sepharose 6 Fast Flow resin (GE Healthcare Life Sciences, Chicago, IL). Chromatographic fractions were analyzed by 18% SDS–PAGE, pooled, and extensively dialyzed against 0.1 M acetic acid (pH 3.0) at 4 °C. Insoluble material was removed by centrifugation and filtration. The resulting fusion constructs were acidified to pH 2.0 with 0.6 M HCl to induce cleavage at the Asp–Pro linker, purged with N_2_, and incubated at 60 °C for 24 h. Subsequently, the pH was adjusted to 7.0–7.2 with 1 M NH_3_ and the mixture incubated overnight at 28 °C to selectively precipitate the onconase carrier, which is insoluble at neutral to alkaline pH. The peptides were recovered from the supernatant by repeated centrifugation and finally lyophilized. In the case of the r­(P)­PRT33 peptide, reverse-phase high-performance liquid chromatography (RP-HPLC) was performed to separate the full-length peptide from truncated fragments, using a Jasco LC-4000 system equipped with PU-4086 semipreparative pumps and an MD-4010 photodiode array detector. A Europa Protein 300 C18 column (5 μm, 25 × 1 cm; Teknokroma, Barcelona, Spain) was employed. The mobile phases consisted of solvent A (0.05% trifluoroacetic acid in water) and solvent B (0.05% trifluoroacetic acid in acetonitrile). The peptide was eluted using the following gradient: isocratic at 5% B for 10 min, 5–20% B over 5 min, 20–35% B over 40 min, 35–45% B over 5 min, 45–95% B over 5 min, followed by isocratic elution at 95% B for 15 min. The flow rate was maintained at 2.0 mL min^–1^, and elution was monitored at 280 nm. In all cases, the purity of recombinant peptides was assessed by 18% SDS-PAGE and mass spectrometry.^104^ The synthetic D­(P)­YLL19 peptide was obtained from CASLO ApS (Kongens Lyngby, Denmark). According to the manufacturer’s analysis by HPLC and mass spectrometry, the peptide was 99.03% pure, with a measured molecular weight of 2537.30 Da.

### Viral Strains

Human coronavirus 229E (HCoV-229E, ATCC VR-740), herpes simplex virus type 1 (HSV-1, strain SC16), and coxsackievirus B3 (CV–B3, strain Nancy, ATCC VR-30) were obtained from the American Type Culture Collection (ATCC, Manassas, VA). Viruses were propagated in Vero-76 cells as previously described.^105,106^ Cell monolayers at approximately 80% confluence were infected at a multiplicity of infection (MOI) of 0.01 in serum-free DMEM for virus amplification. After 1 h of adsorption at 37 °C, the inoculum was removed and replaced with fresh medium containing 2% fetal bovine serum (FBS). Cultures were incubated until the appearance of a cytopathic effect (CPE). Viral supernatants were collected, clarified by low-speed centrifugation (3,000 *g*, 10 min, 4 °C), aliquoted, and stored at −80 °C. Viral titers were determined by plaque assay on Vero-76 cells, yielding stock concentrations of 1 × 10^9^ plaque-forming units (PFU) mL^–1^ for HCoV-229E and HSV-1, and 1 × 10^8^ PFU mL^–1^ for CV-B3.

### Antimicrobial Activity

The antimicrobial activity of MMP-19-derived EPs was assessed against a panel of pathogens, such as *A. baumannii* ATCC 19606, *B. cenocepacia*, *B. multivorans*, *E. faecalis* ATCC 29212, *E. coli* ATCC 25922, *K*. *pneumoniae* ATCC 700603, *S. aureus* ATCC 12600, *P. aeruginosa* 14, *P. aeruginosa* AA2, *P. aeruginosa* ATCC 27853, *P. aeruginosa* KK27, *P. aeruginosa* PA01, *P. aeruginosa* RP73, *S. enteriditis* RIVM 706, *S. enterica* serovar Typhimurium ATCC 14028, *S. aureus* ATCC 29213, methicillin-resistant *S. aureus* MRSA WKZ-2, and *S. epidermidis* ATCC 35984 by using the broth microdilution method.^56^ Bacterial cultures were grown to mid logarithmic phase in Mueller Hinton Broth (MHB) at 37 °C. Cells were then diluted to approximately 2 × 10^6^ CFU mL^–1^ in 0.5× Difco Nutrient Broth (NB, Becton-Dickenson, Franklin Lakes, NJ) and mixed 1:1 (*v/v*) with 2-fold serial dilutions of the peptides (0–80 μmol L^–1^). The use of 0.5× NB was intentionally selected to prevent peptide precipitation and reduce medium-derived interference, as previously reported for cationic AMPs, where nutrient-rich media can attenuate or mask antimicrobial activity. This experimental condition has been consistently adopted in multiple studies by our research group and others, ensuring methodological consistency within this field of research.^107–109^ Following overnight incubation, samples were serially diluted, plated on Tryptic Soy Agar (TSA), and incubated at 37 °C for 24 h to count the number of colonies. Minimal inhibitory concentration (MIC) values were determined as the lowest peptide concentration able to completely inhibit bacterial growth, whereas minimal bactericidal concentration (MBC) values were determined as the lowest peptide concentration able to determine the complete death of bacterial cells. All experiments were carried out in three independent replicates.

### DiSC_3_(5) Assay

Three independent cytoplasmic membrane depolarization assays were carried out on *A. baumannii* ATCC 19606 and *S. epidermidis* ATCC 35984 cells using the 3,3′-dipropylthiadicarbocyanine iodide, DiSC_3_(5) from TCI America, which is a membrane potential-sensitive dye.^110^ Bacterial cells were grown to mid logarithmic phase, then washed and resuspended in 5 mmol L^–1^ HEPES buffer (pH 7.2) containing 0.1 mol L^–1^ KCl and 20 mmol L^–1^ glucose, at a density corresponding to an optical value at 600 nm (OD_600_) of 0.03–0.06. The cell suspension was then incubated with 1 μmol L^–1^ DiSC_3_(5) for 45 min to stabilize the fluorescence, and then the peptides were added to bacterial suspensions at concentrations corresponding to 0.5×, 1× and 2× MIC. Changes in fluorescence intensity were continuously recorded by using GloMax Discover System (Promega, Madison, WI), with excitation and emission wavelengths of 620 and 670 nm, respectively.

### ζ Potential Measurements

To perform analyses, bacteria were grown overnight in MHB medium, then diluted to 2 × 10^8^ CFU mL^–1^ in NB 0.5X and mixed with the peptide under test (1:1 *v/v*) in a final volume of 200 μL. After 15, 30, and 60 min, the bacteria treated with peptide molecules as well as untreated control samples were taken and diluted in 800 μL of NB 0.5X to reach a final volume of 1 mL. The ζ potential of bacterial cells was determined at 25 °C from the mean of 3 independent measurements (30 runs each), in the absence and in the presence of increasing peptide concentrations. ζ potential values were obtained by phase analysis light scattering (PALS) in a Zetasizer Nano ZS 90 device (Malvern, Worcestershire, UK), equipped with Helium–Neon laser (633 nm) as a source of light, with the detection at 173 degree scattering angle at room temperature (25 °C), using disposable Zeta cells with gold electrodes. Values of viscosity and refractive index were set to 0.8872 cP and 1.330, respectively.

### Checkerboard Assay and Definition of Fractional Inhibitory Concentration (FIC) Index

Combinations of MMP-19-derived EPs and antibiotics were tested on *A. baumannii* ATCC 19606 and *S. epidermidis* ATCC 35984 bacterial strains by the so-called “checkboard” assay to determine the FIC indexes. To this purpose, 2-fold serial dilutions of each peptide were tested in combination with 2-fold serial dilutions of antibiotics widely used in topical formulations (*i*.*e*., colistin and polymyxin B). The FIC indexes of the two-drug combinations were calculated as follows: FIC_A_ + FIC_B_, where ${\mathrm{FIC}}_{A}=\frac{\mathrm{MIC A in combination}}{\mathrm{MIC A alone}}$ and ${\mathrm{FIC}}_{B}=\frac{\mathrm{MIC B in combination}}{\mathrm{MIC B alone}}$ . FIC indexes ≤ 0.5 are classified as synergistic, while FIC indexes between 0.5 and 1 or between 1 and 4 are associated with additive and indifferent effects, respectively. Antagonism is instead associated with a FIC index >4.

### Killing Kinetic Studies

To kinetically analyze the antibacterial effects of MMP-19-derived EPs alone or in combination with conventional antibiotics (*e*.*g*., colistin and polymyxin B), experiments were carried out using *S. epidermidis* ATCC 35984 and *A. baumannii* ATCC 19606 treated with a combination of both antimicrobials or with the single agents at concentrations corresponding to their MIC or MBC values. Bacterial cells were diluted to 2 × 10^6^ CFU mL^–1^ in 0.5× NB (Nutrient Broth, Difco, Becton Dickinson, Franklin Lakes, NJ) and mixed at a ratio of 1:1, *v/v* with the peptide, the antibiotic, or both. At defined time points, samples were serially diluted, and each dilution was plated on Luria–Bertani (LB) agar. Following an incubation of 20 h at 37 °C, colonies were counted.

### Bacterial Resistance Development Assay

*A. baumannii* ATCC 19606 and *S. epidermidis* ATCC 35984 bacterial strains were exposed to colistin, polymyxin B, ciprofloxacin, r­(P)­YLL19, r­(P)­YLL33 or r­(P)­PRT33 for prolonged time intervals. Once we detected the MIC values for each peptide or antibiotic against the bacterial strains tested, we transferred bacterial cells that survived into a new well and exposed them to subinhibitory (MIC/2) concentration values of the respective peptide or antibiotic.^56^ The treatment was repeated for 21 days. Strains that developed resistance to the antibiotic under test presented higher MIC values at subsequent passages. The cells from the last passage were isolated and stored for scanning electron microscopy (SEM) analyses.

### Scanning Electron Microscopy Analyses

To carry out SEM analyses, *A. baumannii* ATCC 19606 cells were incubated with 5 μM r­(P)­YLL19, r­(P)­YLL33, and r­(P)­PRT33 peptides, while *S. epidermidis* ATCC 35984 cells were incubated with 5 μM r­(P)­YLL33, 10 μM r­(P)­YLL19, and 10 μM r­(P)­PRT33 peptides. Bacterial suspensions were adjusted at the concentration of 0.1 OD mL^–1^ (corresponding to approximately 1 × 10^8^ CFU mL^–1^) and incubated with peptides for 3 and 16 h at 37 °C. Following incubation, samples were processed and characterized as previously reported.^16^ Briefly, following incubation, bacterial cells were collected by centrifugation at 10,000 rpm for 10 min at 4 °C and subsequently fixed in 2.5% (*v*/*v*) glutaraldehyde. After overnight incubation, the cells were washed three times with distilled water (dH_2_O) and dehydrated through a graded ethanol series: 25% ethanol for 10 min, 50% ethanol for 10 min, 75% ethanol for 10 min, 95% ethanol for 10 min, and 100% anhydrous ethanol for three consecutive 30 min washes. Dehydrated bacterial cells were deposited onto glass substrates and sputter-coated with a thin layer of Au–Pd using a Denton Vacuum DeskV Sputter Coater. Morphological characterization of the samples was performed using a FEI Nova NanoSEM 450 operated at an accelerating voltage of 5 kV, employing both an Everhart–Thornley Detector (ETD) and a Through-Lens Detector (TLD) for high-resolution imaging.

### Anti-Biofilm Activity Assays

Anti-biofilm activity assays were carried out on *S. aureus* ATCC 29213, *S. aureus* MRSA WKZ-2, *S. enteriditis* 706 RIVM, *S. enterica* serovar Typhimurium ATCC 14028, *S. epidermidis* ATCC 35984 and *A. baumannii* ATCC 19606. Bacteria were grown overnight at 37 °C and then diluted to 1 × 10^8^ CFU mL^–1^ in 0.5 × MHB medium. Incubations with increasing concentrations of each peptide (0–40 μM) were carried out, as previously described, for either 4 or 24 h to test peptide effects on cell attachment or on biofilm formation, respectively.^14,57^ Instead, to evaluate the effect of peptides on preformed biofilm, a bacterial biofilm was formed for 24 h at 37 °C and subsequently treated with increasing concentrations of the peptides. Crystal violet assays and confocal laser scanning microscopy (CLSM) analyses in static conditions were carried out according to the method described by Gaglione et al.^14,16^ To evaluate the ability of MMP-19-derived peptides to modulate biofilm composition, *S. epidermidis* ATCC 35984 and *A. baumannii* ATCC 19606 cells were grown overnight in MHB and subsequently diluted to 1 × 10^8^ CFU mL^–1^ in 0.5× MHB containing 2.5 μM of each peptide under investigation. The cultures were incubated for 24 h to assess the peptides’ effects on biofilm formation. Following incubation, colorimetric assays were conducted. Specifically, biofilm protein content was quantified using the Bradford assay, with a standard curve generated from increasing concentrations of bovine serum albumin (BSA). Biofilm carbohydrate content was measured using the Dubois assay, which involved exposing the biofilm to 5% phenol and sulfuric acid (in a 1:2:5 ratio). A glucose-based calibration curve was used to estimate sugar concentrations in the samples. Optical density was measured at 592 nm for the Bradford assay and at 482 nm for the Dubois assay using an automatic plate reader (Benchmark Plus Microplate Spectrophotometer, Bio-Rad, Hercules, CA). All experiments were performed in biological triplicates, and the results of each assay are based on at least two technical replicates.

### Antiviral Activity

The antiviral efficacy of the peptides was evaluated under four distinct treatment conditions: (i) co-treatment, (ii) virus pretreatment, (iii) cell pretreatment, and (iv) post-treatment assays.^111,112^ In the co-treatment assay, Vero 76 cells were simultaneously exposed to the peptide (at noncytotoxic concentrations) and the virus (MOI 0.01 pfu mL^–1^) for 1 h at 37 °C. For the virus pretreatment assay, the peptide was preincubated with the virus (1 × 10^4^ pfu mL^–1^) for 1 h at 37 °C, and then the mixture was applied to the cells for 1 h. In the cell pretreatment assay, the peptide was added to the cells 1 h prior to viral infection. In the post-treatment assay, cells were first infected with the virus and subsequently treated with the peptide for 1 h at 37 °C. Following each treatment, residual virus particles were inactivated using citrate buffer (pH 3.0), and cells were washed with 5% carboxymethylcellulose in complete medium. Following incubations, cell monolayers were fixed with 4% formaldehyde and stained with 0.5% crystal violet. Viral inhibition was quantified by plaque counting, and percent inhibition was calculated by comparing plaque numbers in treated wells to those in the untreated control samples.

### Eukaryotic Cell Culture, Cytotoxicity Assays and Hemolysis Analysis

Human primary dermal fibroblasts (HDF), human immortalized keratinocytes (HaCaT), human epidermoid carcinoma cells (A431), murine Raw 264.7 leukemic monocytes-macrophages, and African green monkey kidney Vero 76 cells were cultured in high-glucose Dulbecco’s modified Eagle’s medium (DMEM) supplemented with 10% fetal bovine serum (FBS), 1% antibiotics (pen/strep), and 1% l-glutamine. Culture medium for Vero 76 cells was also supplemented with 4.5 g L^–1^ glucose. All cell lines were grown at 37 °C in a humidified atmosphere containing 5% CO_2_. Cells were seeded into 96-well plates (100 μL/well) at a density of 3 × 10^3^ cells/well at 24 h prior to treatment, then incubated in the presence of increasing peptide concentrations (0–100 μM) for 24, 48, and 72 h at 37 °C. In the case of Vero 76 cell line, cells were seeded into 96-well plates (100 μL/well) at a density of 2 × 10^3^ cells/well at 24 h prior to treatment, then incubated in the presence of increasing peptide concentrations (from 0.39 to 50 μM) for 24 h at 37 °C. Following treatment with peptides, MTT assays (3-(4,5-dimethylthiazol-2-yl)-2,5-diphenyltetrazolium bromide) were carried out as previously described.^14,16,113^ Briefly, cell culture supernatants were replaced with 0.5 mg mL^–1^ MTT reagent dissolved in DMEM medium without red phenol (100 μL/well). After 4 h of incubation at 37 °C, the resulting insoluble formazan salts were solubilized in 0.04 M HCl in anhydrous isopropanol and quantified using an automatic plate reader spectrophotometer (Benchmark Plus Microplate Spectrophotometer, Bio-Rad, Hercules, California) by measuring the absorbance at 570 nm. Cell viability was expressed as means of the percentage values compared to control untreated cells. Activation of cells by immunological stimuli was obtained by cotreating cells with 1 μg mL^–1^ of LPS from *P. aeruginosa* 10, *E. coli* (O111:B4) or *B. cenocepacia* J2315 (Merck KGaA, Darmstadt, Germany), and with increasing peptide concentrations (5, 10, 20, and 40 μM) for 24 h. The release of hemoglobin from sheep red blood cells (SRBCs) was used as a measure of the hemolytic activity of MMP-19-derived peptides. To perform the assay, SRBCs were diluted in 2% PBS. Aliquots of cells (100 μL) were mixed with equal volumes (100 μL) of serially diluted peptide solutions (ranging from 100 to 0.78 μM). The positive control consisted of SRBCs mixed with 1% SDS (*v/v*, 1:1), representing complete lysis, while the negative control consisted of SRBCs suspended in PBS, indicating no lysis. Following incubation for 30 min at 37 °C, the samples were centrifuged for 3 min at 4 °C. Subsequently, 50 μL of the supernatant from each sample were transferred into a 96-well plate. Absorbance was measured at 405 nm using an automated microplate reader (Benchmark Plus Microplate Spectrophotometer, Bio-Rad, Hercules, CA). The percentage of hemolysis was calculated using the following formula:

$$\mathrm{Hemolysis}\;\left(\%\right)=\frac{[\left({\mathrm{Abs}}_{405\;\mathrm{nm}}\;\mathrm{peptide}-{\mathrm{Abs}}_{405\;\mathrm{nm}}\;\mathrm{negative control}\right)}{\left({\mathrm{Abs}}_{405\;\mathrm{nm}}\;\mathrm{positive control}-{\mathrm{Abs}}_{405\;\mathrm{nm}}\mathrm{negative control}\right)}]\times 100$$

All experiments were conducted with at least biological duplicates, and the results of each assay are representative of a minimum of three independent replicates.

### DCFH-DA Assay

Reactive oxygen species (ROS) quantification was performed using the DCFH-DA (2′,7′-dichlorodihydrofluorescein diacetate) assay, as described by Culurciello et al.^114^ Briefly, 2 × 10^4^ cells were seeded into a 96-well plate and incubated overnight at 37 °C in a 5% CO_2_ atmosphere. The following day, cells were washed with 1× PBS, treated as appropriate, and then incubated with 20 μM DCFH-DA at 37 °C for 40 min. After incubation, fluorescence was measured at excitation/emission wavelengths of 485/532 nm using a multimode microplate reader (Synergy H4 Hybrid Microplate Reader, BioTek Instruments, Inc., Winooski, VT).

### Lipid Peroxidation Analysis

Lipid peroxidation, measured as thiobarbituric acid-reactive substances (TBARS), or malondialdehyde (MDA) equivalents, was evaluated using the thiobarbituric acid (TBA) colorimetric assay, as previously described.^115^ HaCaT cells were seeded into 6-well plates at a density of 2.5 × 10^5^ cells/well. Following treatment, cells were washed with PBS, harvested, and centrifuged at 500 rpm for 5 min. After discarding the supernatant, 0.5 mL of ice-cold 40% trichloroacetic acid (TCA) and 0.5 mL of 0.67% aqueous thiobarbituric acid (TBA) were added to each pellet. The mixtures were heated at 90 °C for 15 min, cooled on ice for 10 min, and centrifuged at 800 *g* for 10 min. The supernatants were collected, and absorbance was measured at 530 nm using a spectrophotometer. TBARS concentration was calculated using a molar extinction coefficient of 1.56 × 10^5^ M^–1^ cm^–1^ and expressed as nmol TBARS *per* 10^6^ cells.

### Cytokine and Nitric Oxide Quantification in RAW 264.7 Cells

The ability of r­(P)­YLL19, r­(P)­YLL33, and r­(P)­PRT33 to modulate cytokine and nitric oxide (NO) production in RAW 264.7 cells was assessed using ELISA (enzyme-linked immunosorbent assay) and Griess assay, respectively. RAW 264.7 cells (2 × 10^4^ cells/well) were seeded into 96-well microtiter plates and incubated overnight at 37 °C in a 5% CO_2_ atmosphere. The following day, the culture medium was replaced with fresh medium containing one of the following treatments: (i) a mixture of r­(P)­YLL19, r­(P)­YLL33, or r­(P)­PRT33 (5, 10, 20, and 40 μM) coincubated with LPSs from *P. aeruginosa* 10, *E. coli*­(O111:B4), and *B. cenocepacia* J2315; (ii) r­(P)­YLL19, r­(P)­YLL33, or r­(P)­PRT33 alone (at the same concentrations); (iii) LPSs alone from the bacterial strains listed above. After 24 h of incubation, cell supernatants were collected and centrifuged at 5,000 rpm for 3 min at room temperature to remove cell debris. TNF-α and IL-6 levels were quantified using immunoassay kits (DuoSet ELISA kits, R&D Systems, Minneapolis, MN) according to the manufacturer’s instructions. Absorbance was measured at 450 nm, using 550 nm as the reference wavelength to correct for optical imperfections of the microplate. Nitrite concentrations, indicative of NO production, were measured using the Griess Reagent Kit for Nitrite Quantitation (Invitrogen, Thermo Fisher Scientific, Waltham, Massachusetts). Briefly, cell supernatants were mixed in equal volumes with N-(1-naphthyl)­ethylenediamine (component A) and sulfanilic acid (component B), forming the Griess reagent. The reaction mixture was incubated for 30 min at room temperature, and absorbance was measured at 548 nm using a 96-well microplate reader (Synergy H4 Hybrid Microplate Reader, BioTek Instruments, Inc., Winooski, VT).

### Skin Abscess Infection Mouse Model

Six-week-old female CD-1 mice (Charles River, stock number: 18679700–022) were anesthetized with isoflurane, and the dorsal skin was shaved. A superficial linear abrasion was made using a sterile needle. A 20 μL aliquot of *A. baumannii* ATCC 19606, grown in LB medium to an optical density at 600 nm (OD_600_) of 0.5 and then washed twice with sterile PBS (pH 7.4; 9391 *g* for 2 min), was applied to the abraded area at a final concentration of 3.67 × 10^6^ CFU mL^–1^. Peptides and antibiotics were diluted in sterile water to their respective MICs and topically applied to the infection site 2 h postinfection. Untreated control mice received 20 μL of sterile PBS. At 2 and 4 days postinfection, animals were euthanized, and the wounded infected skin area was aseptically and uniformly excised. Tissue was homogenized using a bead beater (25 Hz for 20 min), serially diluted 10-fold, and plated on MacConkey agar plates for CFU quantification. Each treatment group included six mice (*n* = 6), with mice housed individually to prevent cross-contamination. Animals were maintained under standard laboratory conditions (12-h light/dark cycle, 22 °C, 50% relative humidity). The study protocol was reviewed and approved by University Laboratory Animal Resources (ULAR) at the University of Pennsylvania (Protocol #806763). Two independent experiments were conducted, each with 3 mice *per* treatment group. Statistical significance was assessed using one-way ANOVA followed by Dunnett’s post hoc test after log_10_ transformation of CFU counts.

### Statistical Analysis

Statistical analyses were performed using GraphPad Prism software. Data are presented as means ± SEM of biological replicates. Statistical significance was determined using a Student’s *t*-test or one-way ANOVA followed by Bonferroni’s or Dunnett’s multiple comparison post hoc tests, with comparisons made against the respective controls. Significance levels are indicated as follows: *p < 0.05, **p < 0.01, ***p < 0.001, or ****p < 0.0001.

## Conclusions

This study provides the first demonstration that human MMP-19 encodes encrypted AMPs with potent, broad-spectrum activity. The identified peptides exhibit robust antibacterial, anti-biofilm, and antiviral effects through membrane-targeting mechanisms that are effective against multidrug-resistant (MDR) pathogens. These activities are accompanied by a significantly reduced potential for resistance development and highly favorable safety profiles, including negligible cytotoxicity, minimal hemolysis, and strong *in vitro* additive effects with last-line antibiotics such as colistin and polymyxin B. Among the identified candidates, the lead peptide r­(P)­YLL19 and its fully D-enantiomeric analogue, D­(P)­YLL19, demonstrated equivalent efficacy *in vitro* and in a murine skin abscess infection model, with D-enantiomerization providing enhanced proteolytic stability without compromising antimicrobial performance. This underscores the utility of d-amino acid substitution as a rational design strategy to enhance pharmacological durability. Furthermore, reported results support a stereochemistry-independent, membrane-disruptive mechanism of action, a hallmark of many effective AMPs. The study also highlights the therapeutic potential of mining the human proteome for encrypted bioactive sequences, offering a reservoir of host-compatible peptides with intrinsic safety advantages. Moving forward, the integration of computational design, proteome mining, and d-amino acid engineering could accelerate the development of AMP-based therapeutics with improved bioavailability and resistance resilience. Taken together, our findings establish r­(P)­YLL19 and D­(P)­YLL19 as promising lead scaffolds for anti-infective development and offer a compelling blueprint for harnessing encrypted peptides and d-amino acid engineering in the ongoing effort to combat antimicrobial resistance.

## Supplementary Material



## Data Availability

The authors declare that the data supporting the findings of this study are available within the paper and its Supporting Information files. Data is available from the corresponding author upon request.

## Notes

Sliding window analysis of the N-terminal antimicrobial region of MMP-19; sliding window analysis of the central antimicrobial region of MMP-19; schematic representation of the acidic hydrolysis of the ONC-r­(P)­PRT33 chimeric construct; analysis by RP-HPLC of the peptide fragments deriving from the hydrolysis of the acid labile bonds present between the sequences of the carrier onconase and of the r­(P)­PRT33 AMP and within the sequence of r­(P)­PRT33 peptide; expected molecular weight values, purity, and experimentally determined molecular weight values for all the purified peptides; analysis of additive combinations of drugs on*A. baumannii* ATCC 19606 strain; MMP-19-derived peptides effects on the ζ potential of treated bacterial cells; MIC and MBC values of colistin, polymyxin B and ciprofloxacin against*A. baumannii*ATCC 19606 and *S. epidermidis* ATCC 35984; MIC values of colistin, polymyxin B, ciprofloxacin, r­(P)­YLL19, r­(P)­YLL33 and r­(P)­PRT33 upon prolonged treatment of*A. baumannii* ATCC 19606; MIC values of colistin, polymyxin B, ciprofloxacin, r­(P)­YLL19, r­(P)­YLL33 and r­(P)­PRT33 upon prolonged treatment of*S. epidermidis*ATCC 35984; analysis of the effects of MMP-19-derived peptides on biofilm main stages; analysis of the effects of MMP-19-derived peptides on the total content of sugars and proteins in biofilm matrix; cytocompatibility of MMP-19-derived peptides on Vero 76 cells; cytocompatibility of MMP-19-derived peptides on Raw 264.7 cells; oxidative cell state analysis; analysis of the ability of D­(P)­YLL19 peptide to act in synergism with conventional antibiotics on*A. baumannii*ATCC 19606 and*S. epidermidis*ATCC 35984 strains; cytocompatibility of synthetic peptide D­(P)­YLL19; hemolytic activity of D­(P)­YLL19 peptide on sheep red blood cells (PDF)

## ¶

RSE S.p.A. – Ricerca Sistema Energetico, Rome, Italy

## Abbreviations

EPs: encrypted peptides
AMPs: antimicrobial peptides
HDPs: host defense peptides
MMP-19: matrix metallopeptidase 19
MS: mass spectrometry
AS: absolute score
MIC: minimal inhibitory concentration
MBC: minimal bactericidal concentration
SEM: scanning electron microscopy
FIC: fractional inhibitory concentration
ζ: ζ potential
CLSM: confocal laser scanning microscopy
PI: propidium iodide
EPS: extracellular polymeric substance
HCoV-229E: human coronavirus 229E
HSV-1: Herpes simplex virus type 1
CVB3: Coxsackievirus B3
IC_50_: 50% inhibitory concentration
CTRL+: positive control
CTRL–: negative control
SD: standard deviation
SRBCs: sheep red blood cells
RAW 264.7: murine macrophages
LPSs: lipopolysaccharides
NO: nitric oxide
TNF-α: tumor necrosis factor-α
IL-6: interleukin-6
HDF: human primary dermal fibroblasts
HaCaT: human immortalized keratinocytes
A431: human epidermoid carcinoma cells
CFU: colony-forming unit
MRSA WKZ-2: methicillin-resistant *S. aureus*
ATCC: American Type Culture Collection
NB: Nutrient Broth
TSA: Tryptic Soy Agar
DiSC_3_-5: 3,3′-dipropylthiadicarbocyanine iodide
MHB: Mueller Hinton Broth
PALS: phase analysis light scattering
LB: Luria–Bertani
BSA: bovine serum albumin
DMEM: Dulbecco’s modified Eagle’s medium
ROS: reactive oxygen species
TBARS: thiobarbituric acid-reactive substances
MDA: malondialdehyde
TBA: thiobarbituric acid
TCA: trichloroacetic acid
ELISA: enzyme-linked immunosorbent assay
ULAR: University Laboratory Animal Resources
