# Supplementary material for: Discovery of Encrypted Peptides in a Human Matrix Metallopeptidase
Source: JACS Au. 2025 Dec 10;6(1):124–43. doi: 10.1021/jacsau.5c00947 (PMC12848717; doi:10.1021/jacsau.5c00947)
Supplement: Supplementary file 1 [file au5c00947_si_001.pdf]

Supporting Information for Publication

Discovery of Encrypted Peptides in a Human Matrix Metallopeptidase

Rosa Gaglione[1,2,^], Martina Schibeci[1,^], Erika Piccolo[1], Rosanna Culurciello[3], Carla Zannella[4], Francesca Mensitieri[5], Fabrizio Dal Piaz[5], Valeria Cafaro[3], Anna De Filippis[4], Elio Pizzo[3], Eugenio Notomista[3], Marcelo D. T. Torres[6-9], Cesar de la Fuente-Nunez[6-9], Angela Arciello[1,2,]*

[1]. Department of Chemical Sciences, University of Naples Federico II, Via Vicinale Cupa Cintia, 26, 80126, Naples, Italy;
[2]. Istituto Nazionale di Biostrutture e Biosistemi (INBB), Via dei Carpegna, 19, 00165, Rome, Italy;
[3]. Department of Biology, University of Naples Federico II, Via Vicinale Cupa Cintia, 26, 80126, Naples, Italy;
[4]. Department of Experimental Medicine, University of Campania "Luigi Vanvitelli", 80138, Naples, Italy;
[5]. Department of Medicine, Surgery and Dentistry, University of Salerno, 84084, Fisciano, Italy;
[6]. Machine Biology Group, Departments of Psychiatry and Microbiology, Institute for Biomedical Informatics, Institute for Translational Medicine and Therapeutics, Perelman School of Medicine, University of Pennsylvania, Philadelphia, Pennsylvania 19104, United States;
[7]. Departments of Bioengineering and Chemical and Biomolecular Engineering, School of Engineering and Applied Science, University of Pennsylvania, Philadelphia, Pennsylvania 19104, United States;
[8]. Department of Chemistry, School of Arts and Sciences, University of Pennsylvania, Philadelphia, Pennsylvania 19104, United States;
[9]. Penn Institute for Computational Science, University of Pennsylvania, Philadelphia, Pennsylvania 19104, United States.

Corresponding Author: Angela Arciello, Department of Chemical Sciences, University of Naples Federico II, Via Vicinale Cupa Cintia, 26, 80126, Naples, Italy; Istituto Nazionale di Biostrutture e Biosistemi (INBB), Via dei Carpegna, 19, 00165, Rome, Italy; orcid.org/0000-0001-8269-6459; Email: anarciel@unina.it

Table of Contents

Figure S1. Sliding window analysis of the N-terminal antimicrobial region of MMP-19……………….**3**
Figure S2. Sliding window analysis of the central antimicrobial region of MMP-19…..……………….**4**
Figure S3. Schematic representation of the acidic hydrolysis of the ONC-r(P)PRT33 chimeric construct………………………………………………………………………………………..**5**
Figure S4. Analysis by RP-HPLC of the peptide fragments deriving from the hydrolysis of the acid labile bonds present between the sequences of the carrier onconase and of the r(P)PRT33 AMP and within the sequence of r(P)PRT33 peptide……………………………………….........................**5**
Table S1. Expected molecular weight values, purity, and experimentally determined molecular weight values are reported for all the purified peptides………………………………………………**6**
Figure S5. Analysis of additive combinations of drugs on *A. baumannii* ATCC® 19606 strain……...**7**
Figure S6. MMP-19-derived peptides effects on the Zeta potential of treated bacterial cells………...**8**
Table S2. MIC and MBC values of colistin, polymyxin B and ciprofloxacin against *A. baumannii* ATCC® 19606 and *S. epidermidis* ATCC® 35984………….………………………………………..…**8**
Table S3. MIC values of colistin, polymyxin B, ciprofloxacin, r(P)YLL19, r(P)YLL33 and r(P)PRT33 upon prolonged treatment of *A. baumannii* ATCC® 19606……………………….............**9**

Table S4. MIC values of colistin, polymyxin B, ciprofloxacin, r(P)YLL19, r(P)YLL33 and r(P)PRT33 upon prolonged treatment of *S. epidermidi*s ATCC® 35984……..…………………….**9**
Figure S7. Analysis of the effects of MMP-19-derived peptides on biofilm main stages...……………………………………………………………………...………………**10**
Figure S8. Analysis of the effects of MMP-19-derived peptides on the total content of sugars and proteins in biofilm matrix……………………………………………………………….………**11**
Figure S9. Cytocompatibility of MMP-19-derived peptides on Vero 76 cells……..………………..**12**
Figure S10. Cytocompatibility of MMP-19-derived peptides on Raw 264.7 cells…….……………**13**
Figure S11. Oxidative cell state analysis…….…………………………………………………...**14**
Figure S12. Analysis of the ability of D(P)YLL19 peptide to act in synergism with conventional antibiotics on *A. baumannii* ATCC® 19606 and *S. epidermidis* ATCC® 35984 strains…….………**15**
Figure S13. Cytocompatibility of synthetic peptide D(P)YLL19……..……………………………**16**
Figure S14. Hemolytic activity of D(P)YLL19 peptide on sheep red blood cells…….……………**17**

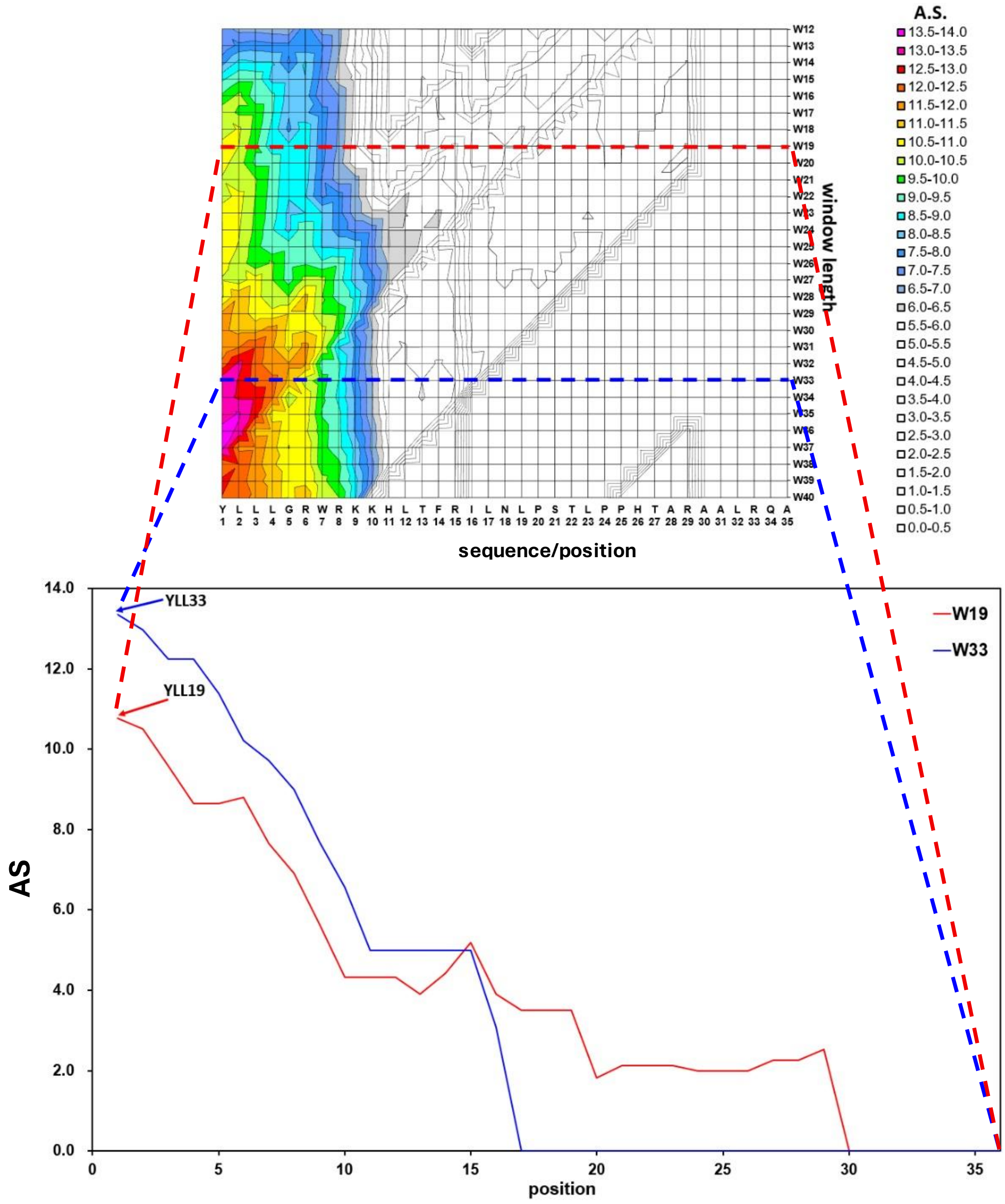

Figure S1. **Sliding window analysis of the N-terminal antimicrobial region of MMP-19.** The isometric plot displays Absolute Score (AS) values (z axis, color scale) as a function of position (x axis) and peptide length (window length, y axis). Each grid node represents a peptide of the specified length starting at the indicated position, and the color of the corresponding region reflects the AS value. The two-dimensional plot shows AS values as a function of position for selected window lengths (19 and 33 residues), which are highlighted by dashed lines in the isometric plots.

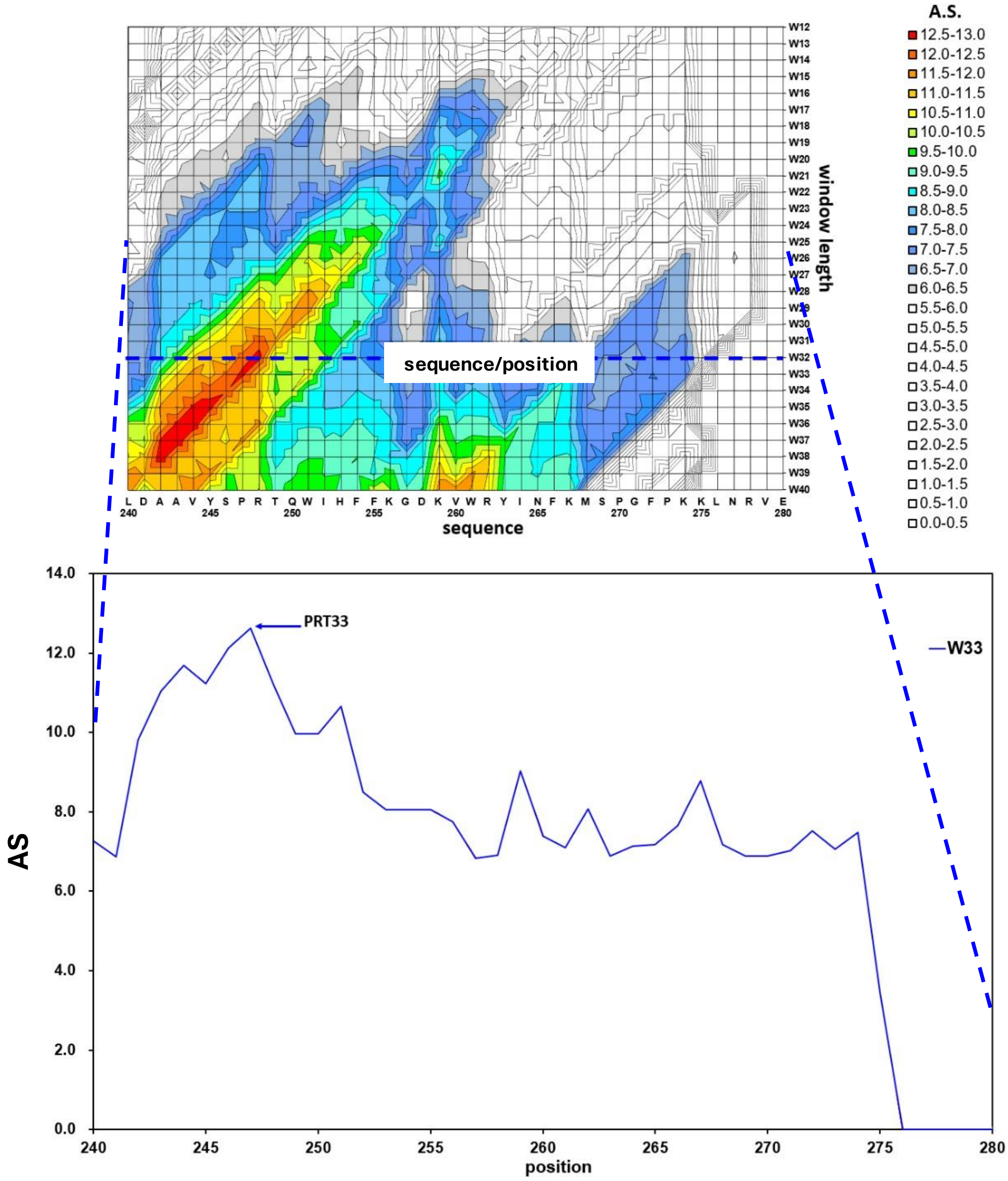

Figure S2. **Sliding window analysis of the central antimicrobial region of MMP-19.** The isometric plot displays Absolute Score (AS) values (z axis, color scale) as a function of position (x axis) and peptide length (window length, y axis). Each grid node represents a peptide of the specified length starting at the indicated position, and the color of the corresponding region reflects the AS value. The two-dimensional plot shows AS values as a function of position for selected window length (33 residues), which is highlighted by dashed lines in the isometric plot.

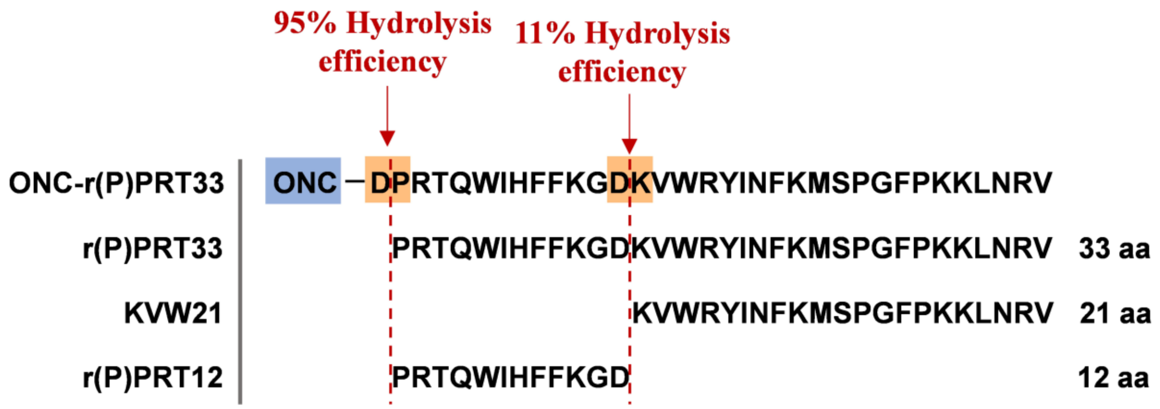

Figure S3. Schematic representation of the acidic hydrolysis of the ONC-r(P)PRT33 chimeric construct. Amino acid sequences of r(P)PRT33 full peptide and of the KVW21 and r(P)PRT12 peptide fragments generated by acidic hydrolysis are reported.

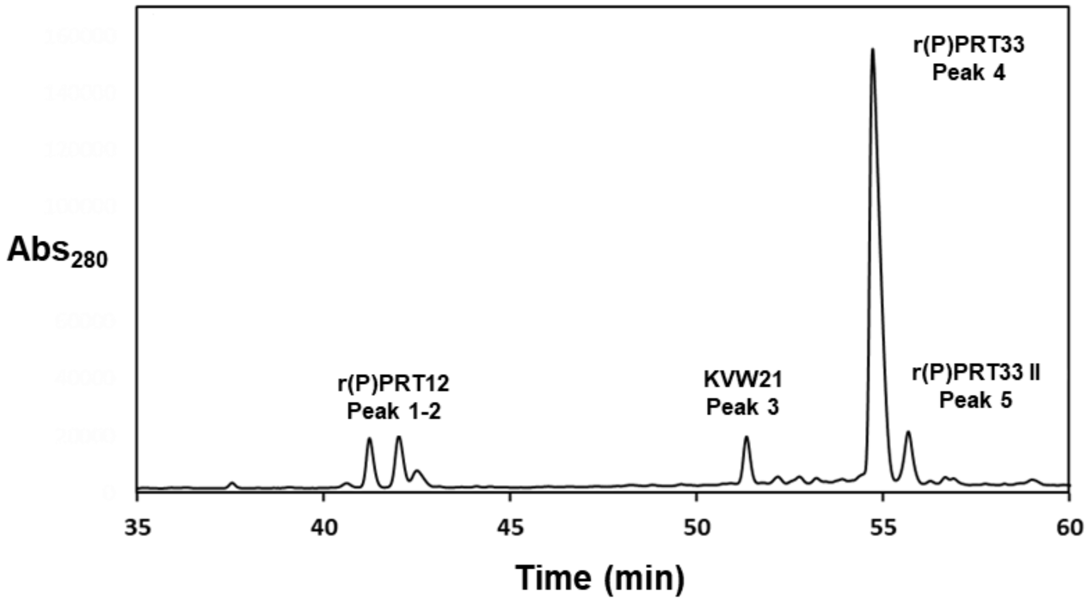

Figure S4. Analysis by RP-HPLC of the peptide fragments deriving from the hydrolysis of the acid labile bonds present between the sequences of the carrier onconase and of the r(P)PRT33 AMP and within the sequence of r(P)PRT33 peptide. The chromatogram was obtained upon selective precipitation of the onconase carrier protein.

Table S1. Expected molecular weight values, purity, and experimentally determined molecular weight values are reported for all the purified peptides.

	PEPTIDE					
	r(P)YLL19	r(P)YLL33	r(P)PRT33 (II)	r(P)PRT33	KVW21	r(P)PRT12
**Expected MW**	**2538.13 Da**	**4007.84 Da**	**4123.23 Da**	**4123.08 Da**	**2609.29 Da**	**1531.8 Da**
**Purity after HPLC**	/	/	**98.7%**	**97.7%**	**97.4%**	**50.2% + 49.8%**
**MW by MS/MS spectrometry**	**2537.53 Da**	**4007.35 Da**	**4123.23 Da**	**4122.23 Da**	**2608.46 Da**	**2608.47 Da + 1530.77 Da**

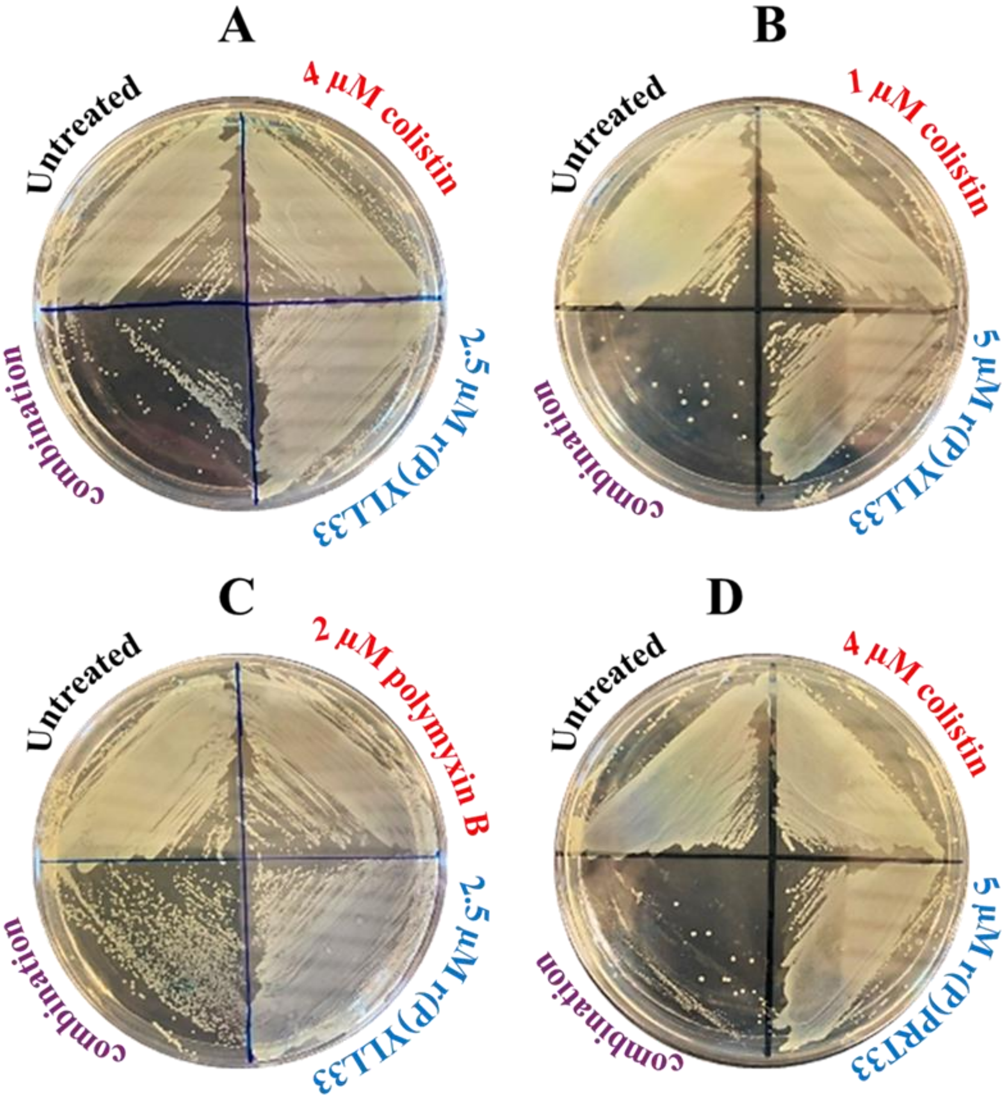

Figure S5. Analysis of additive combinations of drugs on *A. baumannii* ATCC® 19606 strain. **Image A**: effects of a combination of colistin and r(P)YLL33 (**FIC index: 0.750**); **Image B**: effects of a combination of colistin and r(P)YLL33 (**FIC index: 0.650**); **Image C**: effects of a combination of polymyxin B and r(P)YLL33 (**FIC index: 1.0**); **Image D**: effects of a combination of colistin and r(P)PRT33 (**FIC index: 1.0**).

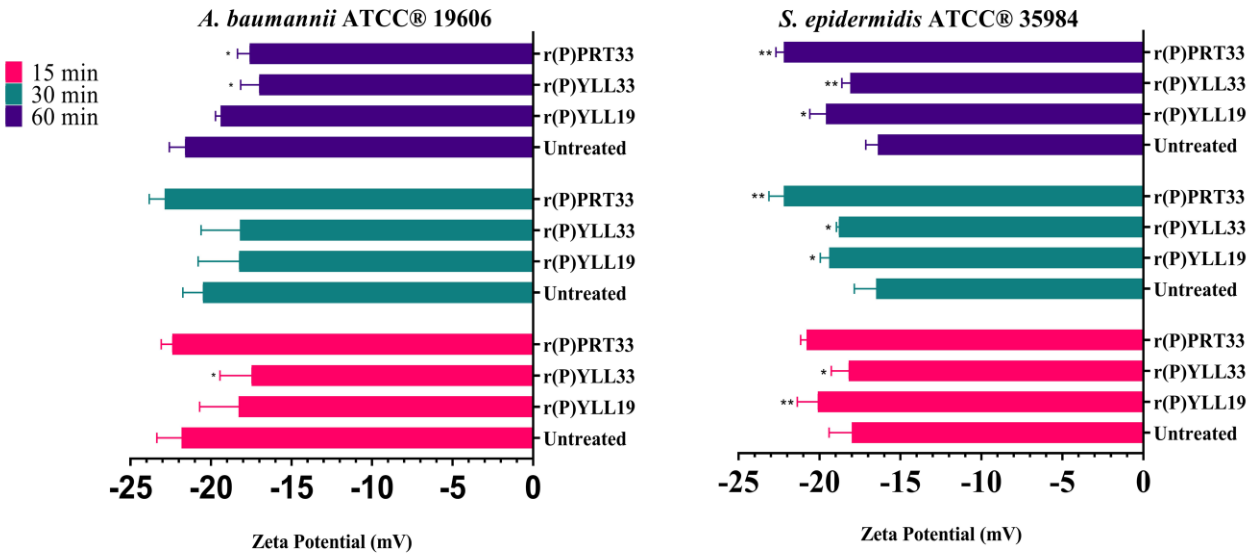

Figure S6. MMP-19-derived peptides effects on the Zeta potential of treated bacterial cells. Zeta potential values of *A. baumannii* ATCC® 19606 and *S. epidermidi*s ATCC® 35984 bacterial strains were determined at different time intervals upon treatment with r(P)YLL19, r(P)YLL33 and r(P)PRT33 peptides at a concentration corresponding to their MIC values. Data represent the average of at least three independent experiments. Statistical analyses were performed using a Student's t-Test. Significant differences were indicated as *($p < 0.05$), **($p < 0.01$) or ***($p < 0.001$).

Table S2. MIC and MBC values of colistin, polymyxin B and ciprofloxacin against *A. baumannii* ATCC® 19606 and *S. epidermidi*s ATCC® 35984.

STRAIN	COLISTIN		POLYMYXIN B		CIPROFLOXACIN	
	MIC	MBC	MIC	MBC	MIC	MBC
A. baumannii **ATCC® 19606**	4 µM	8 µM	2 µM	4 µM	6 µM	6 µM
S. epidermidis **ATCC® 35984**	8 µM	16 µM	4 µM	8 µM	2.5 µM	6 µM

Table S3. MIC values of colistin, polymyxin B, ciprofloxacin, r(P)YLL19, r(P)YLL33 and r(P)PRT33 upon prolonged treatment of *A. baumannii* ATCC® 19606. Values are expressed in µM.

	NUMBER OF TREATMENTS																				
**COMPOUND**	**1**	**2**	**3**	**4**	**5**	**6**	**7**	**8**	**9**	**10**	**11**	**12**	**13**	**14**	**15**	**16**	**17**	**18**	**19**	**20**	**21**
	MIC values (µM)																				
Colistin	4	4	4	4	4	4	4	4	4	4	4	4	4	4	4	4	4	4	4	4	4
Polymyxin B	2	2	2	2	2	2	2	2	2	2	2	2	2	2	2	2	2	2	2	2	2
Ciprofloxacin	**6**	**12**	**12**	**12**	**12**	**12**	**24**	**24**	**24**	**24**	**24**	**24**	**24**	**24**	**24**	**24**	**96**	**96**	**96**	**96**	**96**
r(P)YLL19	5	5	5	5	5	5	5	5	5	5	5	5	5	5	5	5	5	5	5	5	5
r(P)YLL33	5	5	5	5	5	5	5	5	5	5	5	5	5	5	5	5	5	5	5	5	5
r(P)PRT33	5	5	5	5	5	5	5	5	5	5	5	5	5	5	5	5	5	5	5	5	5

Table S4. MIC values of colistin, polymyxin B, ciprofloxacin, r(P)YLL19, r(P)YLL33 and r(P)PRT33 upon prolonged treatment of *S. epidermidi*s ATCC® 35984. Values are expressed in µM.

	NUMBER OF TREATMENTS																				
**COMPOUND**	**1**	**2**	**3**	**4**	**5**	**6**	**7**	**8**	**9**	**10**	**11**	**12**	**13**	**14**	**15**	**16**	**17**	**18**	**19**	**20**	**21**
	MIC values (µM)																				
Colistin	8	8	8	8	8	8	8	8	8	8	8	8	8	8	8	8	8	8	8	8	8
Polymyxin B	4	4	4	4	4	4	4	4	4	4	4	4	4	4	4	4	4	4	4	4	4
Ciprofloxacin	**1.5**	**3**	**12**	**12**	**12**	**24**	**24**	**24**	**24**	**24**	**24**	**48**	**48**	**48**	**48**	**48**	**48**	**48**	**48**	**48**	**48**
r(P)YLL19	5	5	5	5	5	5	5	5	5	5	5	5	5	5	5	5	5	5	5	5	5
r(P)YLL33	5	5	5	5	5	5	5	5	5	5	5	5	5	5	5	5	5	5	5	5	5
r(P)PRT33	5	5	5	5	5	5	5	5	5	5	5	5	5	5	5	5	5	5	5	5	5

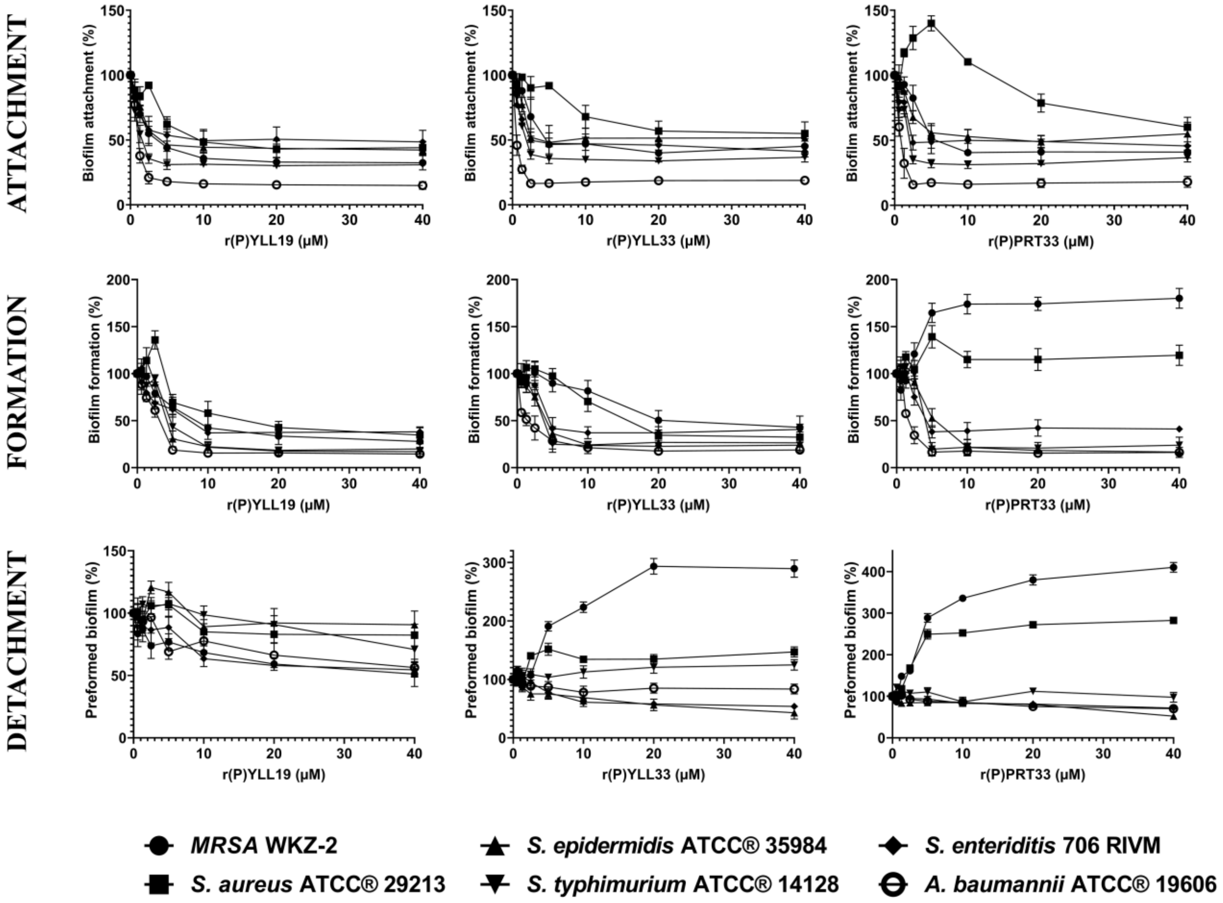

Figure S7. Analysis of the effects of MMP-19-derived peptides on biofilm main stages. The graphs report the data obtained by testing the ability of MMP-19 derived peptides to affect biofilm attachment (**first row**), formation (**second row**) and detachment (**third row**). Selected bacterial strains were treated for different time intervals with increasing concentrations (0–40 µM) of r(P)YLL19, r(P)YLL33 and r(P)PRT33. Anti-biofilm effects of increasing concentrations of each peptide were then tested by crystal violet assay s.

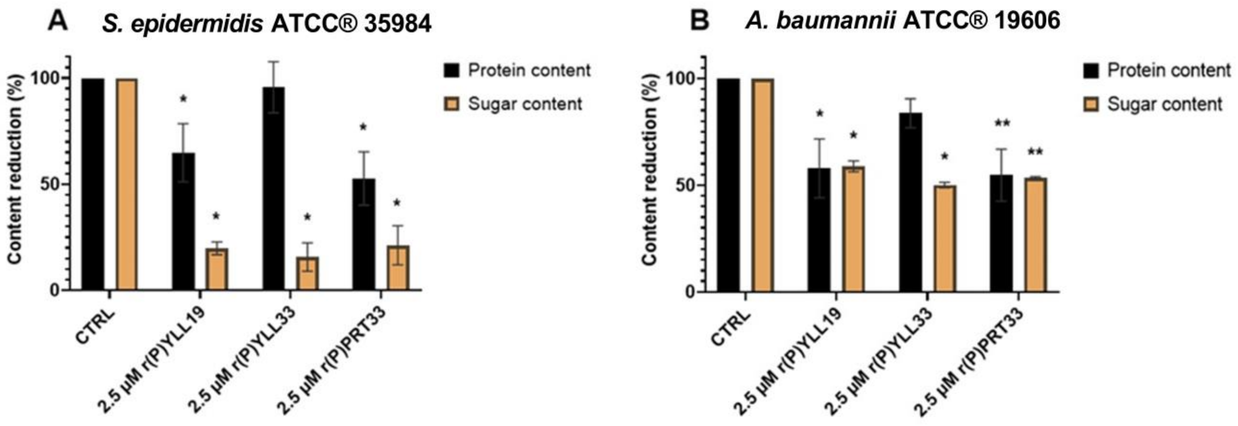

Figure S8. Analysis of the effects of MMP-19-derived peptides on the total content of sugars and proteins in biofilm matrix. The graphs report the sugar content (black bars) and the protein content (orange bars) in the case of control cells and in the case of *S. epidermidis* ATCC® 35894 (A) and *A. baumannii* ATCC® 19606 (B) cells treated with sub-lethal concentrations of peptides under study in experimental conditions known to favour biofilm formation. Total proteins were quantified by Bradford assay while sugars were detected by Dubois assay. Graphs refer to three independent experiments each one performed with triplicate determinations.

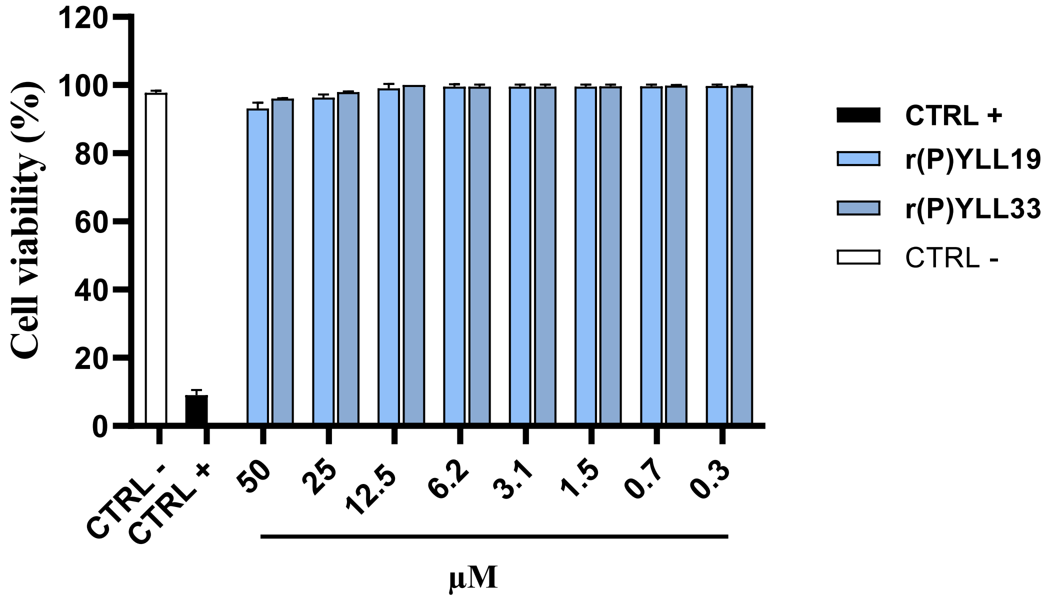

Figure S9. Cytocompatibility of MMP-19-derived peptides on Vero 76 cells. Cell toxicity was assessed by incubating Vero 76 cells with increasing concentrations of the peptides (50 - 25 - 12.5 - 6.2 - 3.1 - 1.5 - 0.7 - 0.3 µM) for 24 h. Cytocompatibility was evaluated by measuring the metabolic activity of viable cells through the reduction of MTT to formazan salts, catalysed by mitochondrial dehydrogenase. Cell viability was expressed as the percentage of viable cells in the presence of the peptide relative to untreated control cells (CTRL-). Cells treated with DMSO served as a positive control for cytotoxicity (CTRL+).

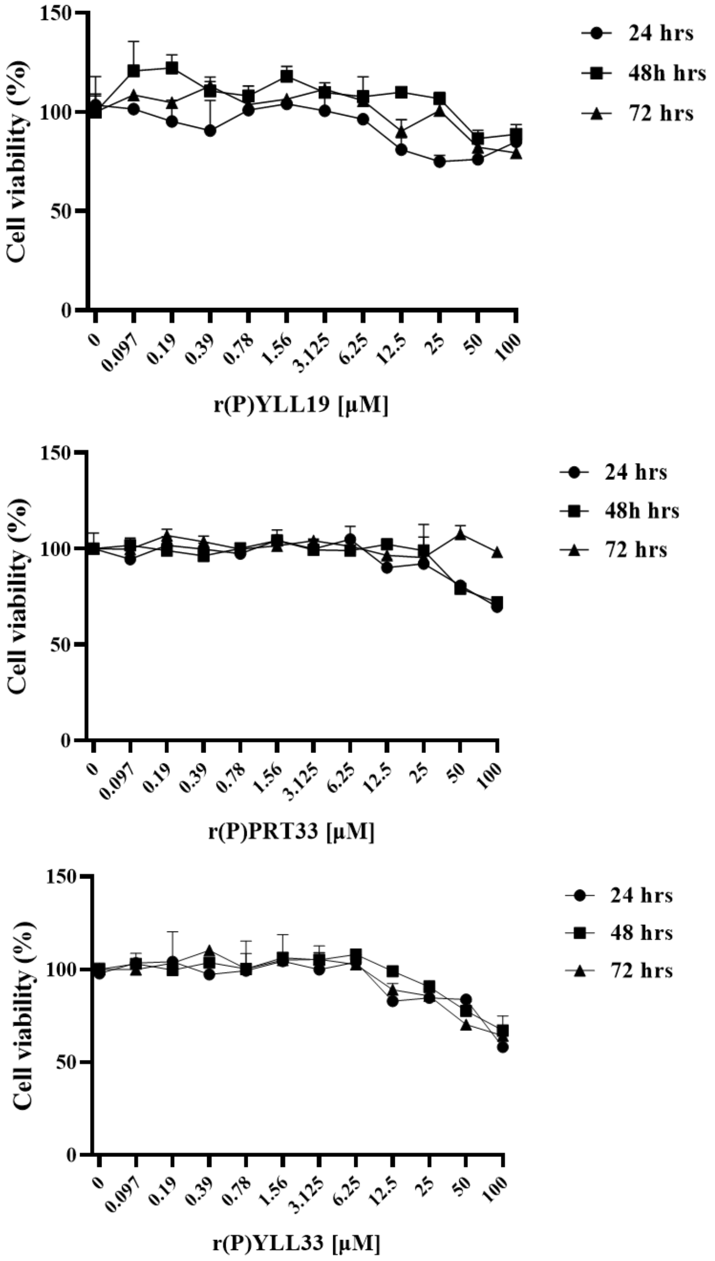

Figure S10. Cytocompatibility of MMP-19-derived peptides on Raw 264.7 cells. Cell toxicity was evaluated by monitoring the metabolic activity of viable cells by quantifying the reduction of MTT to formazan salts due to mitochondrial dehydrogenase. In all cases, cell survival was expressed as the percentage of viable cells in the presence of the peptide under test with respect to control cells grown in the absence of the peptide.

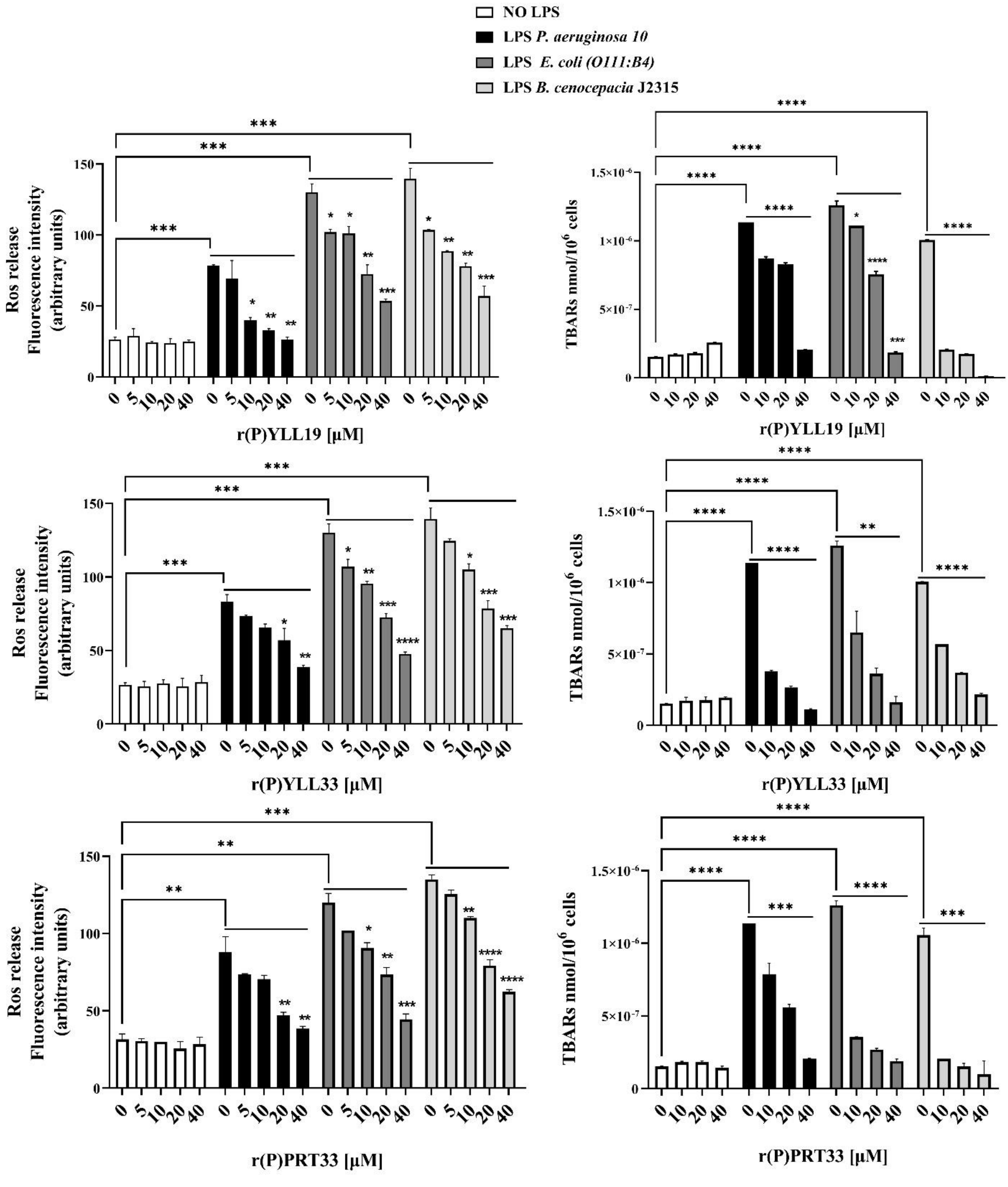

Figure S11. Oxidative cell state analysis. ROS production was analysed by DCFH-Da assay on Raw 264.7 murine macrophage cells that were stimulated with LPSs from *P. aeruginosa* 10, *E. coli* (O111:B4) and *B. cenocepacia* J2315 in co-treatment with r(P)YLL19, r(P)YLL33 or r(P)PRT33. Lipid peroxidation was measured by TBARs assay for r(P)YLL19, r(P)YLL33 or r(P)PRT33. Experiments were performed three times in triplicate determinations and statistical analyses were carried out by using GraphPad Prism (* $p < 0.05$, ** $p < 0.01$, *** $p < 0.001$ or ****$p < 0.0001$).

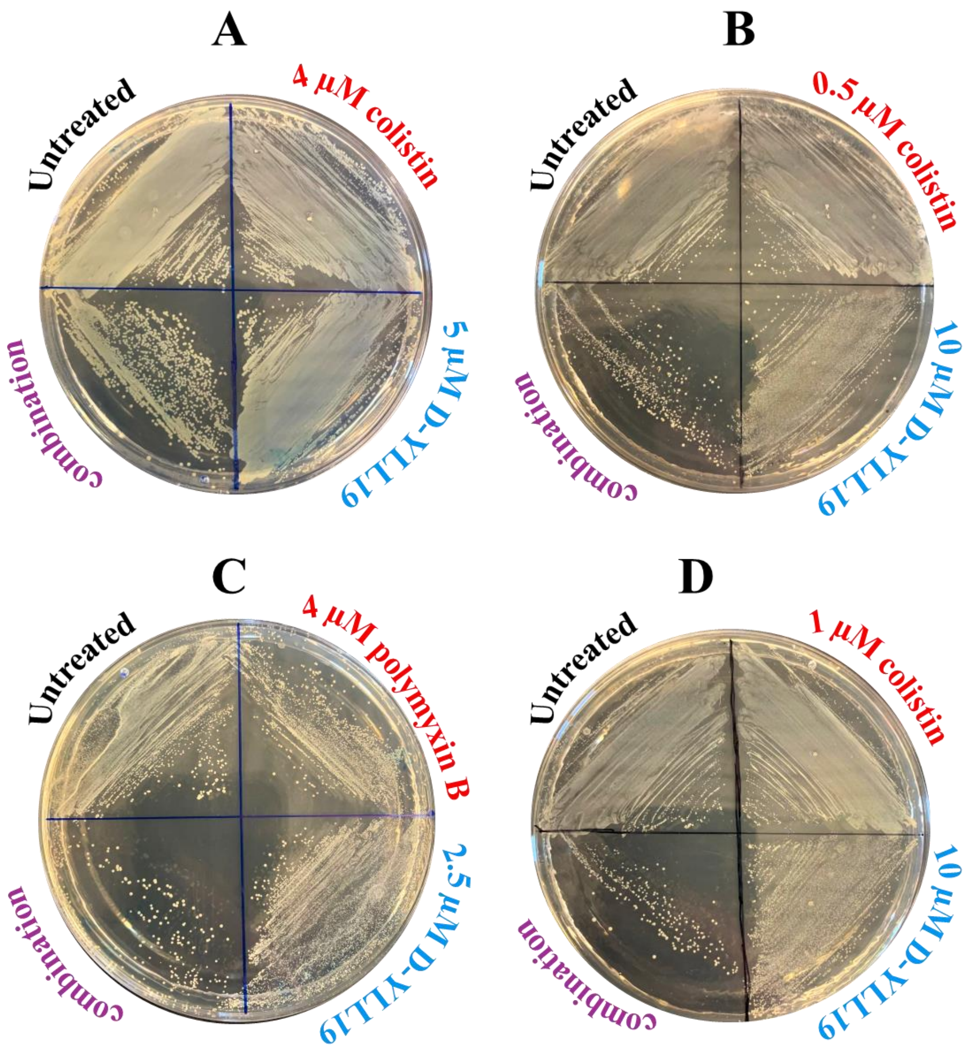

Figure S12. Analysis of the ability of D(P)YLL19 peptide to act in synergism with conventional antibiotics on *A. baumannii* ATCC® 19606 and *S. epidermidis* ATCC® 35984 strains. **Image A**: combination of colistin and D(P)YLL19 against *A. baumannii* ATCC® 19606 (**FIC index: 1.0**); **Image B**: combination of colistin and D(P)YLL19 against *S. epidermidis* ATCC® 35984 (**FIC index: 0.56**); **Image C**: combination of D(P)YLL19 and polymyxin B against *S. epidermidis* ATCC® 35984 (**FIC index: 0.625**); **Image D**: combination of D(P)YLL19 and colistin against *S. epidermidis* ATCC® 35984 (**FIC index: 0.625**).

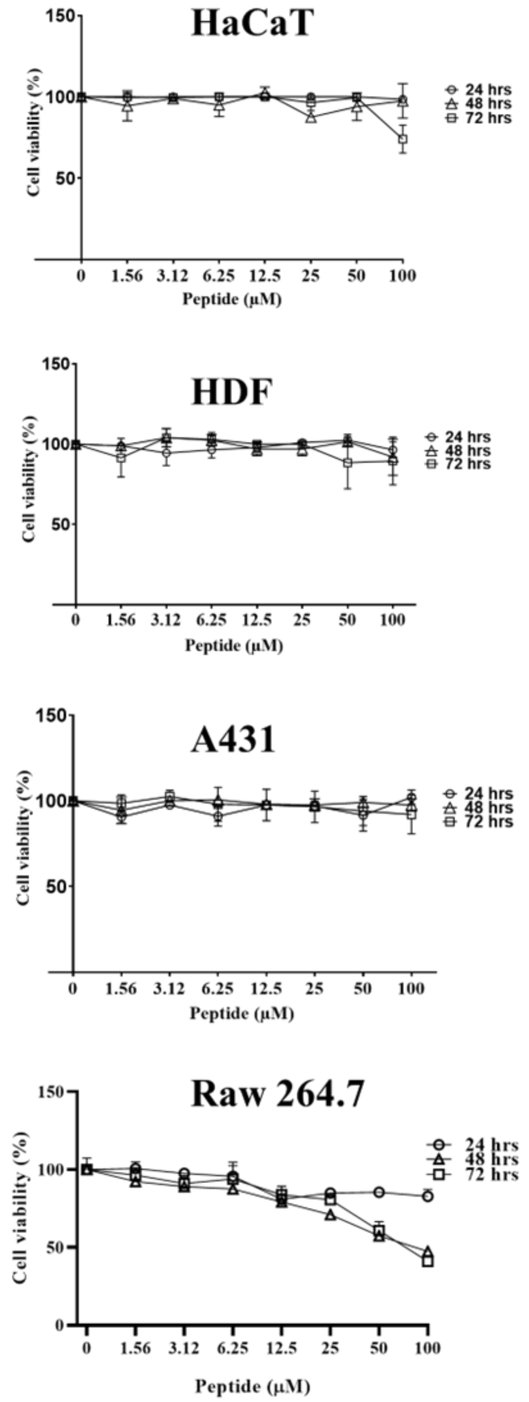

Figure S13. Cytocompatibility of synthetic peptide D(P)YLL19. Graphs report data obtained by treating HaCaT, HDF, A431and Raw 264.7 cells with increasing concentrations of the peptide for 24, 48 and 72 h. In all the cases, cell survival was expressed as the percentage of viable cells in the presence of the peptide under test with respect to control cells grown in the absence of the peptide.

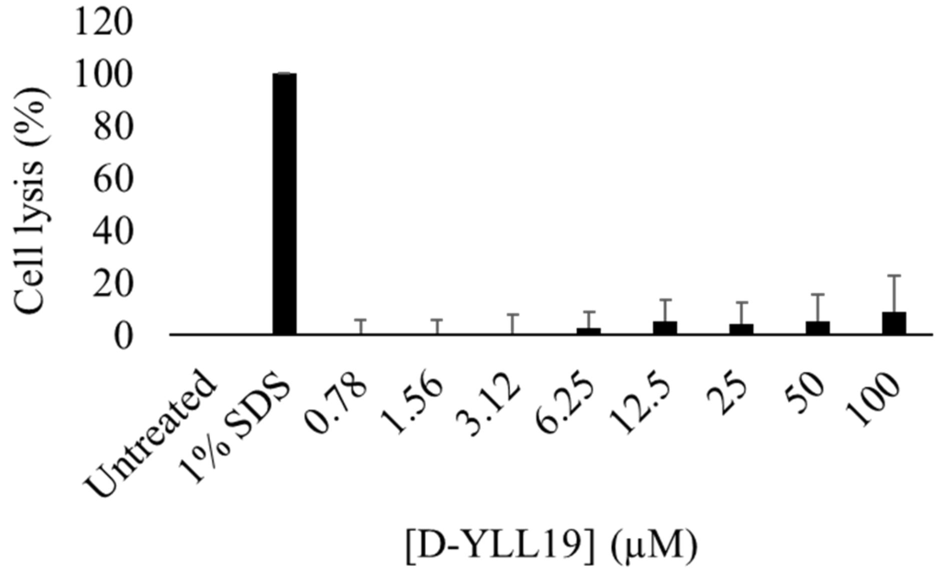

Figure S14. Hemolytic activity of D(P)YLL19 peptide on sheep red blood cells. Graph shows the percentage of cell lysis upon treatment with increasing concentrations of D(P)YLL19 or with SDS 1% (1:1 *v/v*) tested as a positive control. Reported data refer to three independent experiments.
